# Enhancing power system stability by coordinating a wind turbine voltage regulator and lead-lag power system stabilizer using GOOSE optimization

**DOI:** 10.1038/s41598-025-97419-z

**Published:** 2025-04-30

**Authors:** Nader M. A. Ibrahim, Attia A. El-Fergany, Bassam A. Hemade

**Affiliations:** 1 Department of Electrical, Faculty of Technology and Education, Suez University, P.O. Box: 43221, Suez, Egypt; 2 Electrical Power and Machines Department, Zagazig University, Zagazig, 44519 Egypt

**Keywords:** Controller coordination, Doubly fed induction generators, Optimization algorithms, Small-signal stability, Wind turbine integration, Electrical and electronic engineering, Energy grids and networks

## Abstract

Integrating wind energy into power systems can negatively impact stability by reducing oscillation damping. Wind Turbine Voltage Regulators (WT VRs) are designed to manage reactive power and maintain voltage stability; however, they often do not coordinate effectively with Power System Stabilizers (PSS) from synchronous generators (SG). This study utilizes the GOOSE Optimization Algorithm (GOA) to optimize and coordinate the gains of the WT proportional-integral virtual regulator (WT PI-VR) and the SG proportional-integral-type lead-lag PSS (PI-type LL-PSS), to enhance power system stability and performance. The GOA performance compared with the Osprey Optimization Algorithm (OOA) and Particle Swarm Optimizer (PSO). The PI-type LL-PSS performance is compared with proportional-integral-derivative PID-PSS configurations, highlighting its robustness. Testing scenarios include step changes, voltage sags, and three-phase short-circuit faults, using metrics like integral time absolute error, settling time, and standard deviation for robustness evaluation. Statistical analysis shows several benefits from the proposed methodology: (i) A 48.85% stability improvement in coordinating WT PI-VR with PID-PSS using GOA versus OOA, (ii) A 24.40% performance boost with GOA over OOA using PI-type LL-PSS, (iii) A 14.4% enhancement when coordinating WT PI-VR with PI-type LL-PSS compared to PID-PSS, and (iv) A 34.23% performance increase using GOA instead of PSO for coordinating WT PI-VR with PI-type LL-PSS.

**Supplementary Information:**

The online version contains supplementary material available at 10.1038/s41598-025-97419-z.

## Introduction

The large-scale integration of wind turbines (WTs) into modern power system grids presents a significant stability challenge, primarily manifested as reduced system inertia. The rotational inertia provided by the large mass of conventional synchronous generators plays a crucial role in damping low-frequency oscillations. However, doubly fed induction generators (DFIGs), commonly adopted in type-3 WTs, do not provide equivalent inertia support, making the grid more susceptible to frequency fluctuations and instability^1–3^. Although modern WTs adopt advanced control strategies to support local voltage stability, the intermittent nature of wind generation introduces tangible voltage variations, necessitating more rigid control mechanisms. On the other hand, the capability for fault ride-through is crucial for ensuring transient stability, as WTs must actively support reactive power during disturbances^4^.

Given the critical need to increase the amount of renewable energy added for economic and environmental benefits, the large-scale integration of wind energy in modern power grids presents challenges and opportunities for grid stability. Effective coordination and optimization between controllers are vital in ensuring seamless, harmonized operation across all system components. In other words, WT VRs typically play a key role in regulating local voltage by adjusting the reactive power they supply, while PSSs are fundamentally designed to enhance power system oscillation damping, thereby improving overall grid stability. Consequently, the effective coordination between WT VRs and PSSs is essential for reinforcing grid stability. However, achieving such coordination in practice is challenging due to several factors. One notable difficulty arises from the difference in response times—WT VRs typically react much faster than PSSs, which can introduce control inconsistencies. Additionally, the intermittent nature of wind energy further complicates system dynamics. Addressing these challenges necessitates advanced control strategies and optimization techniques to ensure that the synergistic integration of both systems effectively contributes to grid stability^5,6^.

The research on PSS-based controllers aims to improve their performance and integration with other controllers in different electric power systems. Basic optimization employs various algorithms for PSS in either single-machine infinite bus (SMIB) power systems^7–10^ or multimachine power systems (MMPS)^11–14^. Coordinating PSS with other controllers is more sophisticated, requiring advanced mathematical and AI algorithms based on ordinary differential equations (ODEs) in both SMIB^15–18^ and MMPS^19–22^. The inclusion of wind energy introduces differential algebraic equations (DAEs) instead of ODEs, increasing complexity and necessitating a more comprehensive mathematical model. ODEs handle simple dynamic systems, whereas DAEs address complex systems with constraints^23^.

Ref.^24^ suggests coordinating PSS with WT VRs using SMIB and modified SMIB (MSMIB) power systems. Ref.^25^ explores coordinating wind farm power oscillation dampers with the SG PSS using particle swarm optimizer (PSO). Ref.^26^ introduces a coordinated robust control strategy for DFIG WTs with a power oscillation damper (POD) and SGs with a PSS to stabilize power system oscillations. Ref.^27^ examined the coordination of PSS for SGs and POD for static var compensators in systems including WTs.

The stability study examined the coordination among controllers using three system structures with increasing complexity: SMIB, MSMIB, and MMPS. SMIB is used for fundamental studies, MSMIB for detailed scenarios, and MMPS for large-scale systems. SMIB assesses individual generator stability, MSMIB improves single-generator models, and MMPS evaluates the stability of the entire power network. MSMIB offers advantages over MMPS by simplifying the analysis of decreased variable coordination, focusing on specific components, and enabling detailed modeling, particularly with DAEs. Thus, MSMIB is suitable for the preliminary design and evaluation of new algorithms and optimization techniques^28,29^. Considering the control structure, the PI-type LL controller has received significant attention from researchers due to its ability to combine the strengths of both PI and lead-lag compensators. The integral action in the PI controller effectively eliminates steady-state errors and ensures precise setpoint tracking. The lead component enhances phase margin, stability, and response times, directly improving dynamic performance. Additionally, the lag element plays a crucial role in reinforcing the overall resilience of systems against disturbances and parameter variations. These combined characteristics enhance the robustness of the lag element to external changes^30–32^.

A modified Grey Wolf Optimizer technique, as in^33^, optimizes PI-type LL controllers to enhance the power system stability via UPFC-based optimal PI-lead-lag control. In^33^, linearized small-signal stability models were adopted, which fail to capture nonlinear dynamics and fully account for various fault scenarios. Furthermore, the study did not evaluate the coordination between the developed controller and other controllers within the system, thereby limiting its applicability to larger multi-machine power systems. Using the Differential Evolution optimization method, the STATCOM controller employs a PI-type lead-lag controller synchronized with the PSS^34^. A UPFC with a proportional-integral and fractional lead-lag (UPFC-PI-FLL) controller optimized by a whale optimization algorithm improves small-signal stability in systems with varying solar integration^35^. In^35^, the study focused on the coordination of STATCOM with the Differential Evolution (DE) algorithm, without benchmarking against other modern or conventional optimization techniques (e.g., Osprey, PSO), raising concerns about its effectiveness and reliability. Additionally, the study assesses performance considering a narrow range of disturbances and avoids critical scenarios such as three-phase faults and voltage sags, which are widely recognized as essential for evaluating system robustness. Inspired by goose behaviors, the 2024 GOOSE Optimization Algorithm (GOA) enhances the search process and has shown exceptional results in various benchmarks and engineering challenges^36^.

Researchers applied the GOA to optimize a power flow model for IEEE 30-bus and 118-bus networks, incorporating traditional thermal units and renewable energy sources (RESs), addressing operational uncertainties^37^. Ref.^38^ presented an RNN-based technique for monitoring load margins using Graylag Goose Optimization (GGO) to select system buses for RNN input measurements. The challenges of model generalization, the need for extensive training data, prolonged training times, and complications in real-time applications are examined in^38^. The study employs the heuristic optimizer GGO, which demonstrates reasonable performance despite its susceptibility to local optima. However, its analysis is limited to static load variations, which may not be enough for comprehensive stability assessments.

Briefly, this research introduces several key contributions of wind-integrated power system stability:

1. A coordination strategy using GOA is proposed to optimize a WT PI-VR and a PI-type LL-PSS simultaneously. This technique delivers a remarkable 24.40% performance improvement over Osprey Optimization Algorithm (OOA) in coordinating a WT PI-VR and a PI-type LL-PSS.
2. A systematic evaluation of PI-type LL-PSS versus traditional PID-PSS is conducted, specifically in hybrid wind-SG systems. The study emphasizes that integrating WT PI-VR with PI-type LL-PSS compared to PID-PSS leads to a 14.4% enhancement in damping performance.
3. GOA’s effectiveness is rigorously benchmarked against state-of-the-art optimization techniques, including OOA and PSO, ensuring credibility and confidence in the results. The findings demonstrate that employing GOA for coordinating WT PI-VR with PI-type LL-PSS results in a 34.23% improvement in overall performance versus PSO and a 48.85% stability improvement in coordinating WT PI-VR with PID-PSS compared to OOA.
4. Unlike prior studies limited to steady-state variations, this research evaluates the proposed controller across diverse real-world grid scenarios, demonstrating its adaptability and robustness.

There are six sections in this study. The second section discusses modeling the system under study, the significance of coordination, and the earlier work highlighted in the introduction. The optimization procedure is covered in depth in the third section, and the stability analysis is covered in the fourth. The fifth section provides a detailed analysis of the findings and discussion, which informs the conclusions in the following six sections.

## Modelling of the system under study

**Fig. 1:**
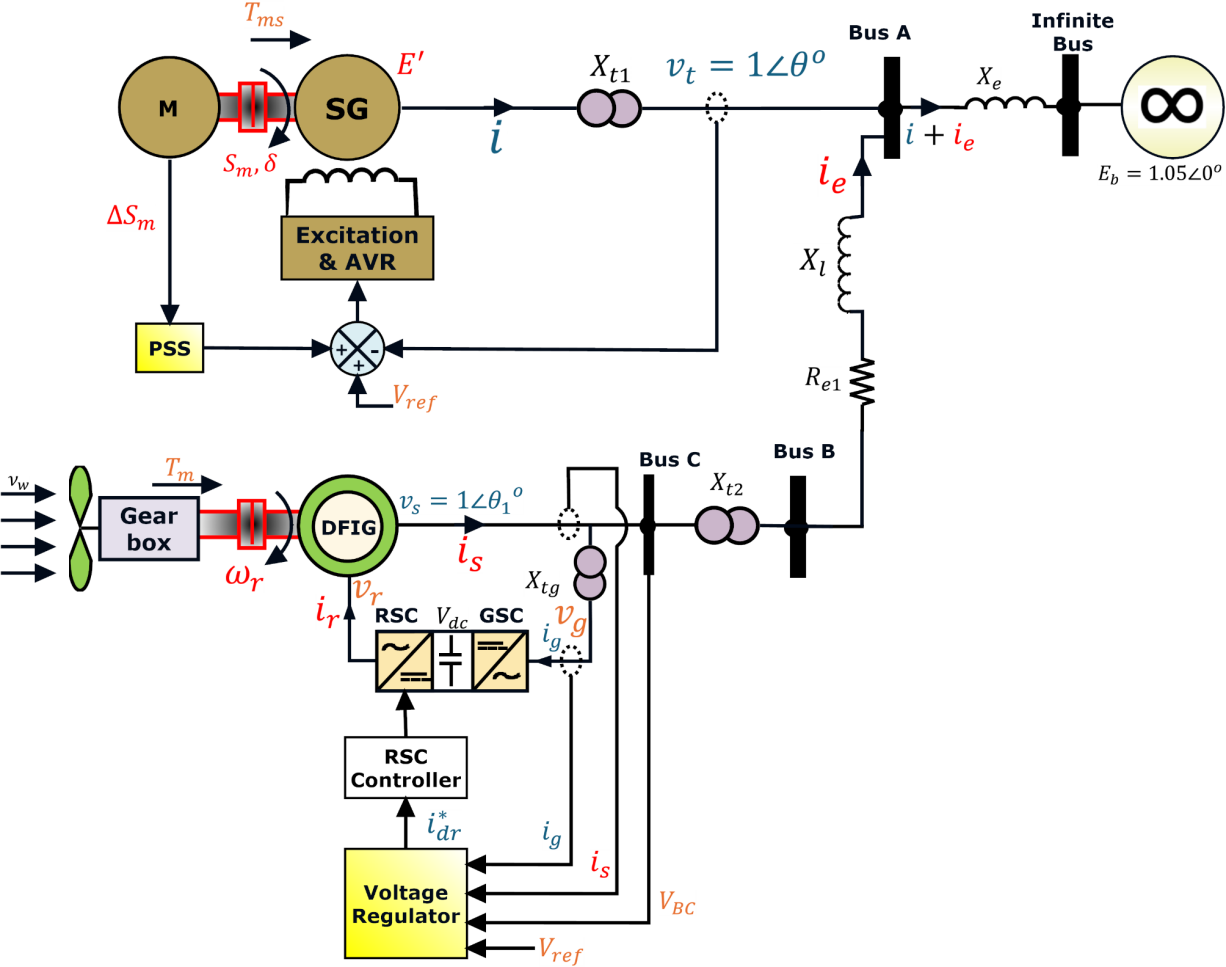
Schematic of MSMIB test system.

This section details the linear model of the MSMIB, which incorporates a type 3 DFIG wind generator linked to the SG and an infinite bus. This model is illustrated in Fig. 1. The proposed model employs a DAE framework that surpasses ODEs for robustness and resilience. DAEs offer a rigorous method for addressing the power system dynamics and algebraic complexities. This enables precise analysis and robust forecasting of system operation and control. However, the following subsections describe the model in detail.

### Generation modeling

The DFIG in the d-q reference frame is derived from three-phase voltage equations. The DFIG dynamics with five state variables and electrical and mechanical torque outputs are summarized in^36–38^. However, fluctuations in wind speed affect the WT performance, which in turn affects their operation. Therefore, the pitch-angle controller is crucial for maintaining the optimal operation by adjusting the blade angles in response to the wind speed, guaranteeing efficient power generation and turbine protection^39,40^.

**Fig. 2:**
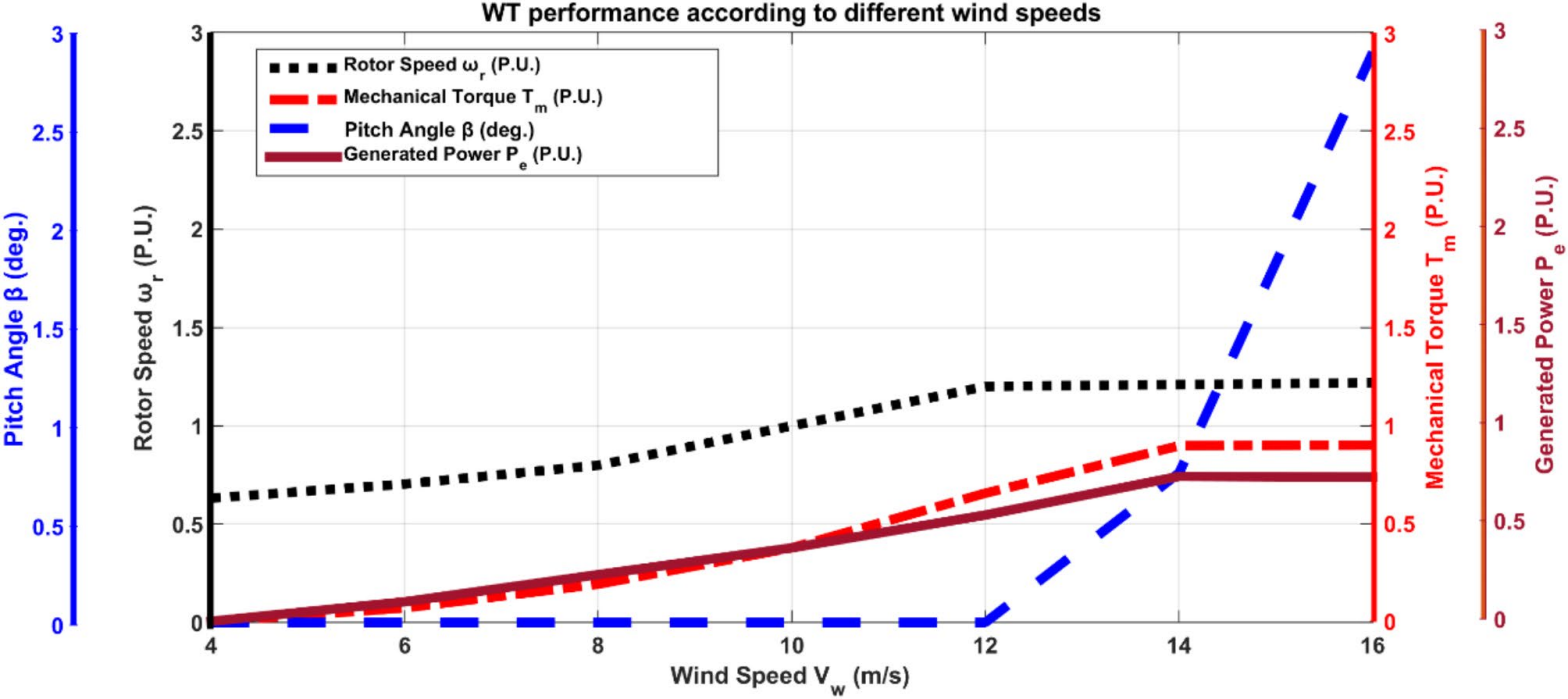
WT performance at different wind speeds.

Figure 2 shows the rotor speed, mechanical torque, mechanical power, and pitch-angle controller response at different wind speeds. The effective power production is between 8 m/s and 12 m/s with a zero-pitch angle, as shown in Fig. 2. The system stability is examined at 12 m/s, representing the maximum wind speed and power generation without the influence of the pitch-angle controller.

The algebraic equations that constrain the dynamic state of the WT and SG can be mathematically formulated as follows^41–43^:

1 $$\:{T}_{e}={L}_{m}({i}_{ds}{i}_{qr}-{i}_{qs}{i}_{dr})$$

2 $$\:{T}_{m}=\frac{0.5\rho\:\pi\:{R}^{2}{C}_{p}{V}_{\omega\:}^{3}}{{\omega\:}_{t}}$$

3 $$\:{v}_{d}=-{X}_{q}{i}_{q}+\varDelta\:{e}_{d}^{{\prime\:}}$$

4 $$\:{v}_{q}={X}_{d}{i}_{d}+\varDelta\:{e}_{q}^{{\prime\:}}$$

5 $$\:{e}_{fd}={K}_{a}({v}_{ref}-\varDelta\:{x}_{1})$$

6 $$\:{\varDelta\:T}_{es}={\varDelta\:e}_{d}^{{\prime\:}}{i}_{d}+\varDelta\:{e}_{q}^{{\prime\:}}{i}_{q}+\left({X}_{d}^{{\prime\:}}-{X}_{q}^{{\prime\:}}\right){i}_{d}{i}_{q}$$

The SG equation model in the DAE framework is studied in detail in^41–43^.

### Interconnection modeling

The remaining system components are also developed in the d-q reference frame, as in^6,27,42,44^. The influence of the SG on Bus A in terms of d-q voltage is summarized in (7) and (8)^6,27,42,44^.

7 $$\:{v}_{d}=-{e}_{bd}\text{sin}\left(\delta\:\right)+{R}_{e}{i}_{d}+{X}_{e}{i}_{q}+{R}_{e}{i}_{de}+{X}_{e}{i}_{qe}$$

8 $$\:{v}_{q}={e}_{bq}\text{cos}\left(\delta\:\right)+{R}_{e}{i}_{q}-{X}_{e}{i}_{d}+{R}_{e}{i}_{qe}-{X}_{e}{i}_{de}$$

In addition, the infinite bus voltage is calculated using (9), and (10)^6,27,42,44^:

9 $$\:{e}_{bd}={e}_{d}-{R}_{e}{i}_{d}+{X}_{e}{i}_{q}$$

10 $$\:{e}_{bq}={e}_{q}-{R}_{e}{i}_{q}-{X}_{e}{i}_{d}$$

The DFIG WT output is connected to the MSMIB via Bus C. However, the influence of WT can be mathematically summarized as follows^6,27,42,44^:

11 $$\:\frac{d{i}_{de}}{dt}=\frac{{\omega\:}_{b}}{{X}_{l}}{v}_{ds}-{\omega\:}_{b}{i}_{qe}-\frac{{\omega\:}_{b}{R}_{e1}}{{X}_{l}}{i}_{de}-\frac{{\omega\:}_{b}}{{X}_{l}}{v}_{d}$$

12 $$\:\frac{d{i}_{qe}}{dt}=\frac{{\omega\:}_{b}}{{X}_{l}}{v}_{qs}+{\omega\:}_{b}{i}_{de}-\frac{{\omega\:}_{b}{R}_{e1}}{{X}_{l}}{i}_{qe}-\frac{{\omega\:}_{b}}{{X}_{l}}{v}_{q}$$

In reference to Fig. 1, d-axis and q-axis stator voltages can be derived by applying Kirchhoff’s Voltage law (KVL), as represented in (13) and (14). In addition, by applying KCL at the DFIG stator output, GSC d- and q-axis currents ($\:{i}_{dg}$ and $\:{i}_{qg})$ can be calculated as in (14) and (15), whereas Eq. (16) represents the current fed into the GSC ($\:{i}_{g}$)^41,44,45^.

13 $$\:{v}_{ds}={v}_{dg}+{X}_{tg}{i}_{qg}$$

14 $$\:{v}_{qs}={v}_{qg}-{X}_{tg}{i}_{dg}$$

15 $$\:{i}_{dg}={i}_{ds}+{i}_{de}$$

16 $$\:{i}_{qg}={i}_{qs}+{i}_{qe}$$

17 $$\:{i}_{g}=\frac{({v}_{g}-{v}_{s})}{{X}_{tg}}$$

It is worth mentioning that the Jacobian matrix, which is essential for analyzing and simulating DAE system models, is derived and thoroughly investigated in^41,44,45^. Appendix (A) also contains the MSMIB power system data, which comprises DFIG, SG, and transmission lines.

### The modeling of controller considering coordination

This study focuses on an in-depth analysis of the coordination between the WT VR and SG PSS as a key aspect of power system stability enhancement. Accordingly, the following subsections will provide an overview of the fundamental structure of WT VR and PSS, followed by a critical examination of their coordinated impact on the system’s DAEs and state-space model.

#### WT voltage regulator

**Fig. 3:**
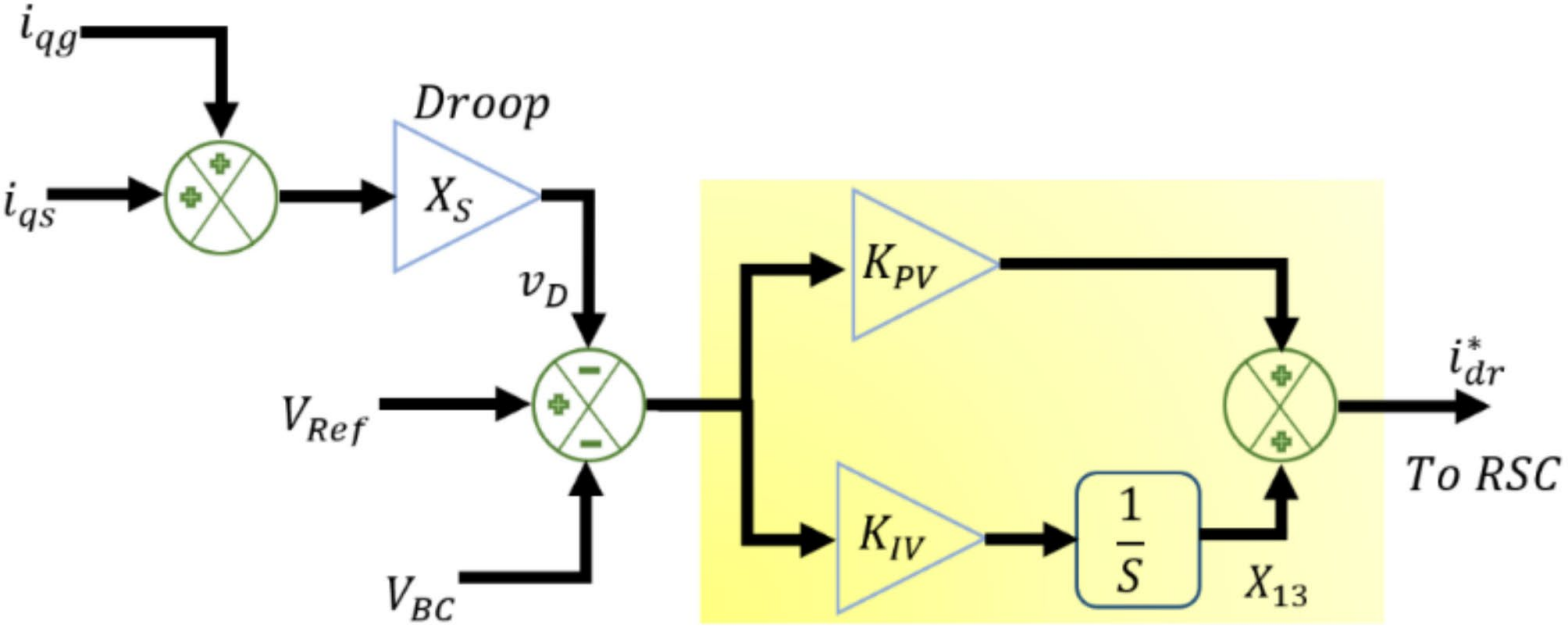
WT voltage-control regulator structure.

Typically, the WT PI-VR loop maintains the desired terminal voltage by regulating the generator excitation based on feedback, which is crucial for reliable and efficient WT power generation. Figure 3 shows the fundamental structure of the WT PI-VR loop adopted in this study. The Figure depicts the controller with three inputs: (1) reference voltage $\:{V}_{Ref}$ (2) voltage magnitude $\:{v}_{D}$ and (3) voltage at Bus C $\:{v}_{BC}$. The interrelations between those inputs are detailed in (18) and (19)^46,47^.

18 $$\:{v}_{D}={X}_{S}({i}_{qS}+{i}_{qg})$$

19 $$\:{v}_{BC}=\frac{1}{3}({v}_{a}+\left({v}_{b}{e}^{\left(j*2*\frac{pi}{3}\right)}\right)+\left({v}_{c}{e}^{\left(-j*2*\frac{pi}{3}\right)}\right)$$

The control structure of the WT voltage controller is represented in (20)^47,48^.

20 $$\:{i}_{dr}^{*}=({V}_{Ref}-{v}_{D}-{v}_{BC})({K}_{PV}+\int\:{K}_{IV})$$

The gains of both controllers (WT VR and PSS of SG) are simultaneously optimized using the OOA and GOA, applied separately to compare their effectiveness in tuning the control parameters. Furthermore, the performance of both algorithms was compared with that of a PSO-optimized controller, using the gains obtained through PSO as a benchmark. The output current of the voltage regulator acted as the input signal to the rotor side converter (RSC) controller. The output voltage of the current regulator can be calculated using rotor current as in (21)^47,48^.

21 $$\:{v}_{d}^{{\prime\:}}=({i}_{dr}^{*}-{i}_{dr})({K}_{PI}+\int\:{K}_{II})$$

where $\:{v}_{d}^{{\prime\:}}$ is the voltage output signal of the current regulator, and $\:{i}_{dr}$ represents the actual direct-axis WT rotor current. The modified control signal ($\:{v}_{d}^{\text{*}}$) added to the RSC input is derived in (22)^47–49^.

22 $$\:{v}_{d}^{*}={v}_{d}^{{\prime\:}}+{R}_{r}{i}_{dr}-\left[{i}_{qr}\left({L}_{lr}+{L}_{m}\right)\left({\omega\:}_{0}-{\omega\:}_{r}\right)\right]-\left[{i}_{qs}{L}_{m}\left({\omega\:}_{0}-{\omega\:}_{r}\right)\right]$$

#### PSS controller

**Fig. 4:**
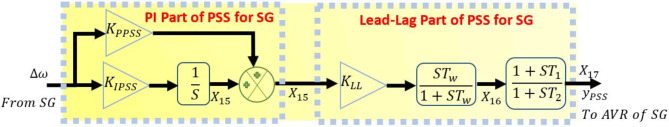
Basic structure of the proposed PI-type lead-Lag-PSS.

The proportional-integral-type lead-lag control structure is adopted for the PSS (PI-type LL-PSS). The gains of both controllers, WT PI-VR and PI-type LL-PSS, have been simultaneously optimized to coordinate their operation. However, the matrix (A) size due to incorporating PI-type-LL-PSS and PI-VR controllers is (17 × 17). The fundamental structure of the proposed PI-type LL-PSS of the SG is illustrated in Fig. 4.

The implemented control structure of the PSS controller is a Proportional-Integral Lead-Lag (PI-type LL-PSS). Unlike a standard PI controller, which only provides proportional and integral actions, the PI-type LL-PSS includes a Lead-Lag compensator that enhances phase compensation for effective oscillation damping. The structure of the implemented PSS consists of the following components:

- PI Controller ($\:{K}_{PPSS},\:\:\&\:{K}_{IPSS}$) – Enhances low-frequency response and eliminates steady-state error.
- Gain Block ($\:{K}_{II}$) – Adjusts the magnitude of the stabilizing signal.
- Washout Filter ($\:{T}_{W}$) – Ensures that only dynamic signals pass through, preventing steady-state drift.
- Lead-Lag Compensator ($\:{T}_{1},\:\:\&\:{T}_{2}$) – Provides phase lead/lag to ensure proper damping of oscillations.

The transfer function of the PI-type LL-PSS implemented in this study is given by^33–35^:

23 $$\:{y}_{PSS}={K}_{II}\times\:\frac{{K}_{PPSS}+{K}_{IPSS}/S}{1+{ST}_{W}}\times\:\frac{1+S{T}_{1}}{1+S{T}_{2}}$$

Additionally, the performance of the PI-type LL-PSS is examined against the proportional-integral-derivative (PID-PSS) to assess its robustness. However, the combination of PID-PSS and PI-VR controllers results in an increase in the size of matrix (A) from (12 × 12) to (15 × 15).

## Goose optimization algorithm

The GOA has demonstrated exceptional performance across benchmark tests and real-world applications, including welded beam design and economic load dispatch^36^. Even though GOA offers versatility and effectiveness compared to other mature optimization algorithms, its computational complexity and scalability should be carefully considered when applied to large-scale problems. In this study, GOA is employed to optimize the gains of both controllers simultaneously, and its performance is systematically evaluated against the OOA and PSO. The optimization function of the GOA can be mathematically formulated as follows^50^:

24 $$\:{\mathbf{X}}_{Gi}\left(t+1\right)={\mathbf{X}}_{Gi}\left(t\right)+\left(\alpha\:\left({\mathbf{X}}_{Gbest}\left(t\right)-{\mathbf{X}}_{Gi}\left(t\right)\right)\right)+\left(\beta\:\cdot\:\left({\mathbf{X}}_{Gj}\left(t\right)-{\mathbf{X}}_{Gi}\left(t\right)\right)\right)+\left(\gamma\:\left({\mathbf{X}}_{Gk}\left(t\right)-{\mathbf{X}}_{Gi}\left(t\right)\right)\right)$$

This equation balances the exploitation of the optimal location by exploring new positions based on the behavior of other geese. This includes principal step equations governing the GOA. The algorithm’s main steps can be summarized as follows:

### Initialization

#### Initialize the population

The geese population is randomly positioned within the search space, as in (25). Initial parameters such as balancing and guarding coefficients are established, generating diverse solutions^50^.

25 $$\:{\mathbf{X}}_{Gi}\left(0\right)={\mathbf{X}}_{\text{Gmin}}+\text{rand}({\mathbf{X}}_{\text{Gmax}}-{\mathbf{X}}_{\text{Gmin}})$$

#### Evaluation

The fitness of each goose is evaluated based on an objective function, which determines the quality of each solution. The fitness of each goose is recorded.$\:\:F\left({\mathbf{X}}_{Gi}\right(t\left)\right)$.

#### Identifying the best position

The best position among the initial positions is determined in (26)^50^.

26 $$\:{\mathbf{X}}_{Gbest}\left(0\right)=\text{a}\text{r}\text{g}\underset{{\mathbf{X}}_{Gi}(t+1)}{\text{m}\text{i}\text{n}}f\left({\mathbf{X}}_{Gi}\right(0\left)\right)$$

### Rest-phase

#### Update positions

Each goose updates its position according to the rest-phase Eq. (27)^50^.

27 $$\:{\mathbf{X}}_{Gi}(t+1)={\mathbf{X}}_{Gi}\left(t\right)+\left(\beta\:\cdot\:\left({\mathbf{X}}_{Gj}\left(t\right)-{\mathbf{X}}_{Gi}\left(t\right)\right)\right)+\left(\gamma\:\left({\mathbf{X}}_{Gk}\left(t\right)-{\mathbf{X}}_{Gi}\left(t\right)\right)\right)$$

#### Evaluation of new positions

The fitness of new positions is stored in$\:\:\:f\left({\text{X}}_{Gi}\right(t+1\left)\right)$.

#### Update the best solution

Equation (28) mathematically defines the process for identifying and updating the optimal solution during the rest-phase^50^.

28 $$\:{\mathbf{X}}_{Gbest}(t+1)=\text{a}\text{r}\text{g}\underset{{\mathbf{X}}_{Gi}(t+1)}{\text{m}\text{i}\text{n}}F\left({\mathbf{X}}_{Gi}\right(t+1\left)\right)$$

### Foraging positions

#### Update positions

Equation (29) represents how each goose updates its position through the foraging phase^50^.

29 $$\:{\mathbf{X}}_{Gi}(t+1)={\mathbf{X}}_{Gi}\left(t\right)+\left(\beta\:\cdot\:\left({\mathbf{X}}_{Gj}\left(t\right)-{\mathbf{X}}_{Gi}\left(t\right)\right)\right)+\left(\gamma\:\left({\mathbf{X}}_{Gk}\left(t\right)-{\mathbf{X}}_{Gi}\left(t\right)\right)\right)$$

#### Evaluation of new positions

The fitness of the new positions is stored in$\:\:\:f\left({\mathbf{X}}_{Gi}\right(t+1\left)\right)$.

#### Update the best solution

Identify and update the best solution for the foraging phase using as in (28).

### Termination check

Evaluating whether the termination or convergence criteria have been met to determine whether the search process should continue or stop:

$$\:if\:t\ge\:Max.-Iter.\:or\:convergence\:criterion\:met,\:stop.$$

### Output

The best solution is the output. Decide whether to continue the search. The final optimized solution is then provided. The optimal solution is reported in$\:\:{\mathbf{X}}_{Gbest}$.

**Fig. 5:**
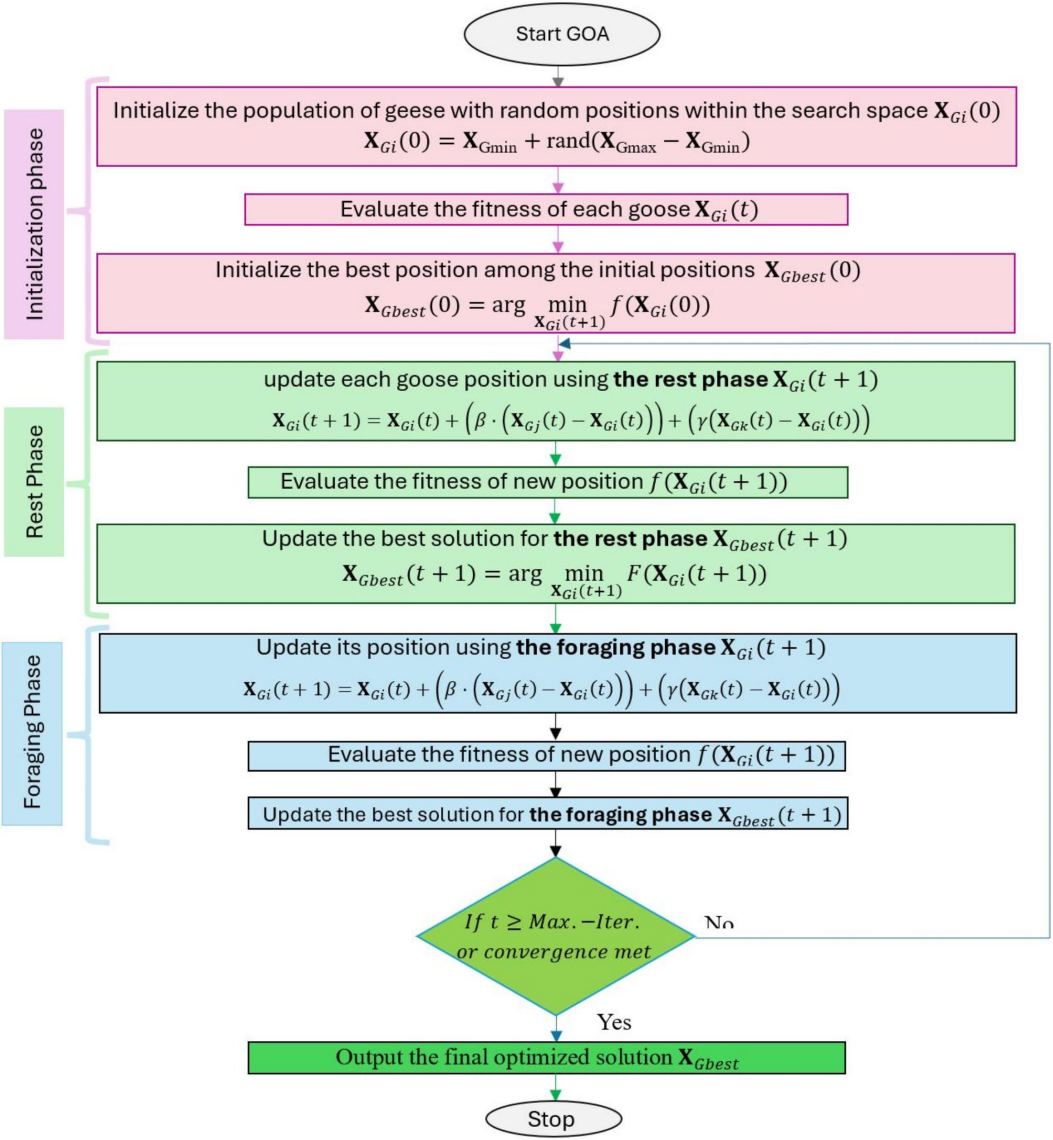
GOA flowchart.

**Fig. 6:**
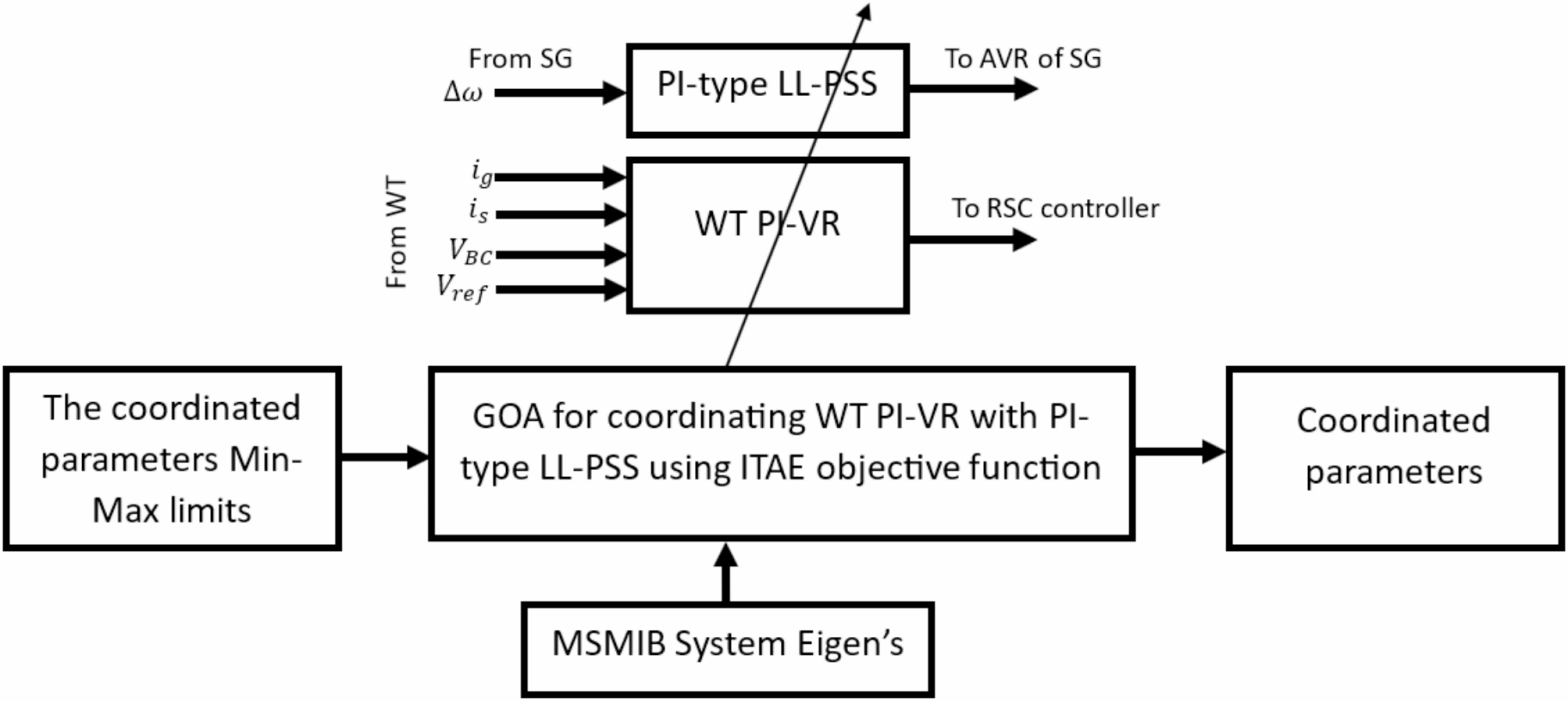
The GOA coordination process.

Figure 5 shows a flowchart representing the GOA steps applied in the coordination process^50^. When coordinating the WT PI-VR with PI-type LL-PSS, the controller tuning process optimizes seven parameters ($\:{K}_{PV}$, $\:{K}_{IV}$, $\:{K}_{PPSSLL}$, $\:{K}_{IPSSLL}$, $\:{K}_{LL}$, $\:{T}_{1}$, and $\:{T}_{2}$). While coordinating the WT PI-VR with the PID-PSS process includes optimizing five parameters ($\:{K}_{PV}$, $\:{K}_{IV}$, $\:{K}_{PPSS}$, $\:{K}_{IPSS}$, and $\:{K}_{DPSS}$). Optimization aims to minimize the integral time absolute error (ITAE) objective function in (30). Figure 6 depicts the coordination process of WT PI-VR with PI-type LL-PSS by GOA using the ITAE as the objective function.

Notably, this study uses two fitness functions in the optimization process, which are defined in (30) and (31)^49,51,52^.

30 $$\:ITAE={\int\:}_{0}^{Ts}t\times\:\left|e\left(t\right)dt\right|\:$$

31 $$\:F1={\rho\:}_{1}\sum\:_{i=1}^{N}{\left({\sigma\:}_{0}-{\sigma\:}_{i}\right)}^{2}+{\rho\:}_{2}\sum\:_{i=1}^{N}{\left({\xi\:}_{0}-{\xi\:}_{i}\right)}^{2}with\:\:{\sigma\:}_{i}\ge\:{\sigma\:}_{0}\:and\:{\xi\:}_{i}\le\:{\xi\:}_{0}$$

Integrating the absolute error over time, the ITAE prioritizes errors, as shown in (30), which promotes faster error correction and effectively minimizes sustained oscillations. The ITAE is offering superior performance in improving settling time and overall system stability compared to the integral square of the error (ISE) or integral absolute of the error (IAE) performance indices^49,51,52^.

## Analysis of system behavior with coordinated controllers

**Fig. 7:**
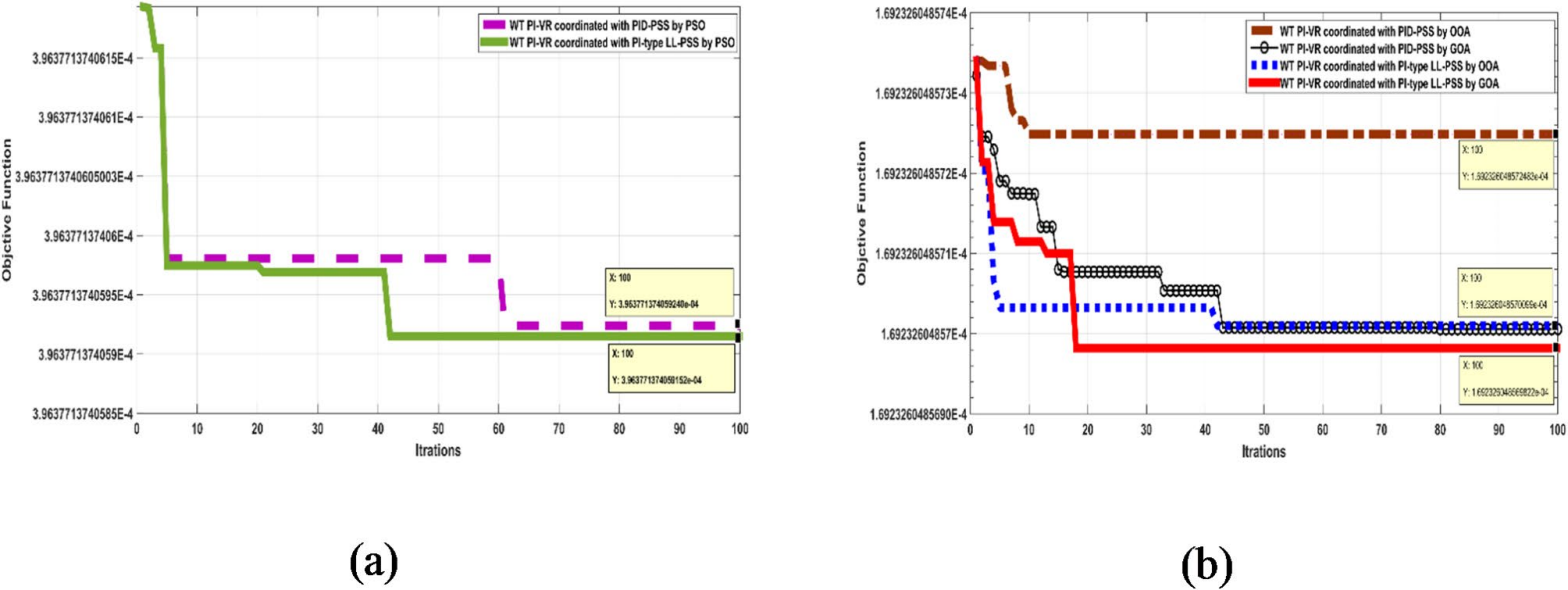
Convergence curves of coordinating different controllers by, (**a**) PSO and (**b**) OOA, and GOA, respectively.

The behavior of both controllers based on matrix A is discussed in this section. Table 1 presents the coordinated gains for the WT PI-VR and PSS controllers. These gains were obtained using the selected optimization algorithms (PSO, OOA, and GOA). Figure 7a illustrates the convergence performance of the fitness functions for the PSO-optimized WT PI-VR coordinated with different PSS controller structures. The convergence performance of the coordinated controller highlights the superiority of the PI-type LL-PSS compared with the PID-PSS controller.

Even though the system matrix with the PI-type LL-PSS was larger than that with the PID-PSS, the PI-type LL-based controller converged much faster than the PID-based controller. Therefore, PI-type LL will be adopted for further evaluation. Additionally, Fig. 7b illustrates the convergence performance of the fitness function with optimized gains using the OOA and GOA optimization algorithms. The convergence performance indicates that the GOA has the fewest iterations when searching for optimal gains compared with the OOA to coordinate both controllers simultaneously. These findings emphasize the superiority of GOA over OOA. Furthermore, the PI-type LL-based controller outperformed the PID controller.

**Table 1:** Coordinated controller values using the OOA and GOA.

Coordinated controllers	Gains	Coordination		
		PSO	OOA	GOA
WT PI-VR coordinated with PID-PSS	$\:{K}_{PV}$	8.9436	10.2502	5.8504
	$\:{K}_{IV}$	555.2425	327.4495	237.0494
	$\:{K}_{PPSS}$	50.5410	70.3520	67.0637
	$\:{K}_{IPSS}$	7.5691	13.3675	12.3332
	$\:{K}_{DPSS}$	6.5502	28.1449	11.4290
WT PI-VR coordinated with PI-type LL-PSS	$\:{K}_{PV}$	10.0592	5.7670	4.8111
	$\:{K}_{IV}$	452.4428	299.8456	283.6976
	$\:{K}_{PPSSLL}$	495.8259	487.2713	472.8259
	$\:{K}_{IPSSLL}$	72.3906	48.0506	42.3906
	$\:{K}_{LL}$	0.0882	0.0489	0.0447
	$\:{T}_{1}$	0.2197	0.1586	0.1197
	$\:{T}_{2}$	0.0372	0.293	0.0272

It is worth mentioning that the GOA achieves optimal gains approximately 57% faster and is 57% more accurate than the PSO algorithm. These findings indicate that the GOA is more efficient and reliable than the PSO in the problem domain.

The following sections discuss the analysis performance for the two coordinated controllers based on the selected optimization algorithms, considering eigenvalues, natural and damped frequencies, synchronizing and damping torques, damping ratio, and participation factor percentage.

Regardless of the optimization algorithm adopted, its application of the optimization algorithm resulted in three oscillatory modes and 11 stationary modes. However, in contrast to uncoordinated controllers, the coordination of WT PI-VR with PID-PSS based on OOA resulted in the movement of three oscillatory modes from unstable to stable regions based on the participation factor analysis. The first oscillatory mode, $\:{\lambda\:}_{\text{5,6}}$ has a frequency of 4 Hz. The $\:{\lambda\:}_{\text{5,6}}$ mode incorporates the WT current and voltage regulator states. The second mode, $\:{\lambda\:}_{\text{7,8}}$, has a frequency of 0.8 Hz, which is related to the PID-PSS state with the d-axis rotor current of the WT. The third mode, $\:{\lambda\:}_{\text{11,12}}$, has a frequency of 1.0025 Hz and is associated with the participation of the d-q axis current that appears through the integration of the WT with the MSMIB. Similarly, applying the GOA resulted in the transition of three oscillatory modes to stable regions based on the eigenvalue analysis of states. This consistency in the states is observed when utilizing the OOA and GOA algorithms. Coordinating WT PI-VR with a PI-type LL-PSS is beneficial, increasing the number of states while maintaining system stability with a lower average natural frequency. The coordinated behavior of both controllers shifts the three oscillatory modes to a stable region, as in the case of the PID-based controller. The first oscillatory mode, $\:{\lambda\:}_{\text{3,4}}$, with a frequency of 1 Hz, was directly related to the WT d-q axis rotor current. The second state, $\:{\lambda\:}_{\text{5,6}}$, has a frequency of 0.2 Hz and represents the incorporation effect of the SG speed deviation and the first state of the PI-type LL-PSS. Furthermore, the third oscillatory state, $\:{\lambda\:}_{\text{7,8}}$, with a frequency of 1.0002 Hz, represents the connection between the SG rotor angle deviation and the second state of the PI-type LL-PSS.

**Fig. 8:**
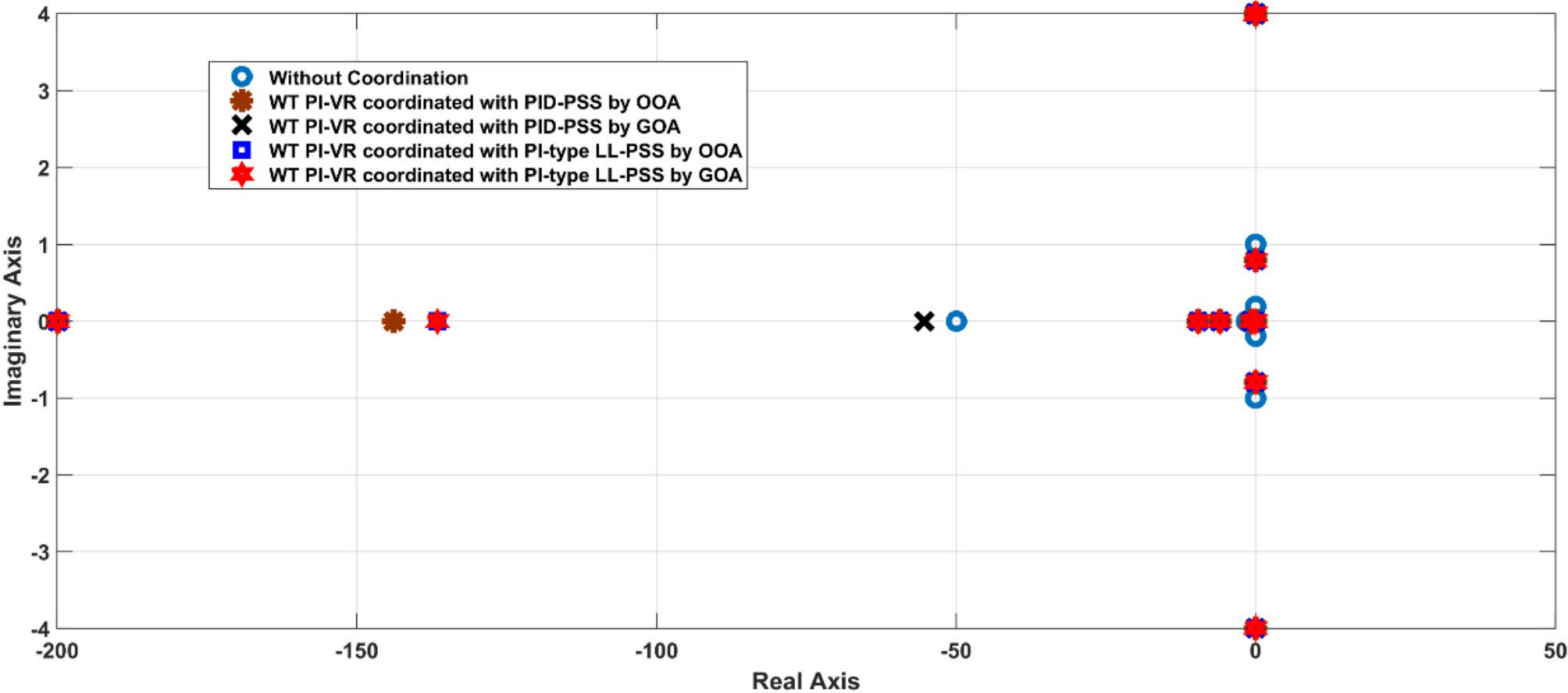
The eigen plot of the system with and without coordination.

The participation factor percentage analysis highlights the influence of oscillatory modes associated with the PI-type LL-PSS and the contributions from both machines. This analysis highlights the significance of simultaneously coordinating both controllers over independently optimizing each controller’s gains. Figure 8 illustrates the system eigen plot with and without the coordination of both controllers. Eigenvalue analysis is crucial for power system stability studies, as it reveals the system’s damping characteristics and dynamic response. Accordingly, Fig. 8 illustrates the distribution of eigenvalues in the complex plane for various controller configurations. Eigenvalues with negative real parts indicate stability, while positive real parts indicate instability. The imaginary part reflects oscillatory behavior, with larger magnitudes indicating lower oscillation frequencies. Irrespective of the controller structure, the results demonstrate that the coordinated tuning of the WT and PSS significantly enhances overall system stability.

Additionally, the eigen plot overviews the evidence that the GOA-optimized PI-type LL-PSS with WT PI-VR shifts the dominant eigenvalues further into the left-half complex plane, increasing system damping and enhancing stability. In contrast, the non-optimized, and PID-PSS configurations result in eigenvalues closer to the imaginary axis. This performance indicates less effective damping and prolonged oscillations. These results confirm that the proposed coordination strategy effectively mitigates oscillatory modes, which ensure a more stable power system operation. Additionally, the coordinated optimization of WT PI-VR with a PI-type LL-PSS is more effective in improving the system stability than the PID-PSS, regardless of the algorithm adopted.

It is worth mentioning that the dominance of non-oscillatory modes over oscillatory modes provides several advantages; (1) it accelerates the system stabilization, which enables the system to achieve stability more rapidly after a disturbance and reduces the recovery time. (2) The reduced likelihood of sustained oscillations ensures a steady and more reliable system response. (3) By suppressing sustained oscillatory behavior, non-oscillatory modes enhance the energy dissipation efficiency of the system, which in turn contributes to improved operational robustness and overall efficiency.

## Results and discussions

Several objective functions are commonly used in power system stability and control applications like IAE, ITAE, ISE, and ITSE. In^53^, the mathematical representation of the four functions has been discussed in detail. Additionally, the analysis conducted in^53^ emphasizes ITAE’s superiority over other performance indices (e.g., IAE, ISE, and ITSE). Unlike IAE, which only accounts for the absolute error, ITAE weights errors over time, ensuring that sustained deviations are heavily penalized. Compared to ISE, which squares the error signal and may amplify brief large deviations, ITAE prioritizes reducing long-term fluctuations, leading to smoother system dynamics. ITSE, while incorporating a time-weighted squared error, can sometimes emphasize later-stage errors excessively, affecting initial transient performance. By effectively balancing transient and steady-state performance, ITAE ensures faster settling time, lower oscillation amplitudes, and enhanced damping characteristics, making it ideal for optimizing controllers like PSS, AVR, and FACTS devices in dynamic power system environments. Consequently, the ITAE has been adopted in this study due to its effectiveness in minimizing overshoot, reducing steady-state error, and enhancing overall system damping.

**Fig. 9:**
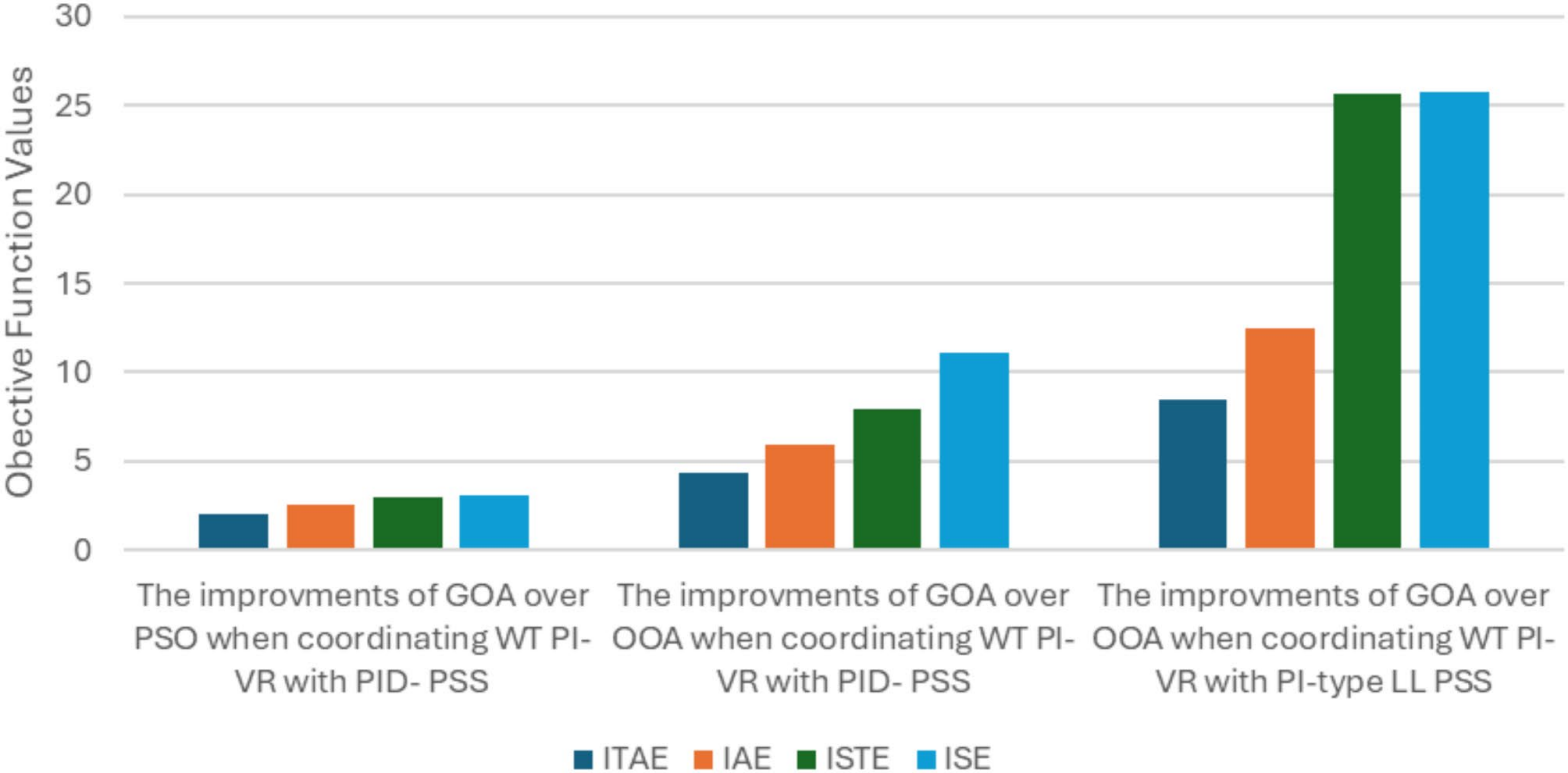
Computed objective functions for GOA over OOA and PSO.

Figure 9 illustrates a comparison of the ITAE, IAE, ISTE, and ISE objective functions. As can be seen, the GOA excels in other optimization algorithms. The results demonstrate that the aim of ITAE function has the lowest value among the other various functions.

**Fig. 10:**
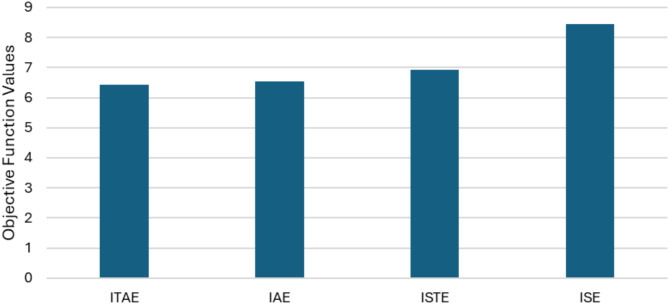
Computed objective functions for different coordination configurations.

Moreover, Fig. 10 presents a comparative analysis of the objective functions, highlighting the advantages of coordinating WT PI-VR with PI-type LL-PSS over PID-PSS. The results indicate that the ITAE achieves the lowest value, demonstrating its effectiveness. Therefore, it can be concluded that the ITAE objective function consistently meets the error minimization goal across all aspects.

The robustness of the proposed GOA-based technique was achieved through two testing scenarios under two operating conditions, as reported in Tables 2 and 3. Table 2 outlines the test scenarios used to examine system stability with and without the optimized PSS. Each scenario is evaluated at two operating points, as listed in Table 3. These scenarios are designed to test five coordinated control structures across two operating points. Additionally, the weighting coefficients $\:{\rho\:}_{1}$ and $\:{\rho\:}_{2}$ The improved parameters for each part of *F*1 are selected as 0.6 and 0.8, respectively. The improved parameters’ minimum and maximum are 0.001 and 500, respectively. The GOA parameters are listed in Table 4.

**Table 2:** Summary of applied test scenarios.

Test scenarios	Test type	Disturbance type (% or P.U. or no. of phases or m/s)	Duration (stat – clear) time or no. of cycles	Location
No. 1	Load step change	30%	From 4 to 4.1 s. (for 0.1 s.)	At bus (A)
No. 2	Short circuit	Three-phase	From 4 to 4.1 s (6 cycles)	At the middle of the TL

**Table 3:** Overview of operating points.

Operating point	Loading	Values
No. 1	Nominal loading	$\:P=0.75\:p.u.,\:Q=0.0588\:p.u.$
No. 2	Heavy loading	$\:P=0.9503\:p.u.,\:Q=0.09\:p.u.$

**Table 4:** GOA parameters.

Parameter	Value
Population size	35
Number of iterations	100
Balance factor	0.5
Foraging behavior	0.7
Resting behavior	0.3
Orthogonal learning mechanism	0.8

Test scenarios from Table 2 are applied according to the Nordic Analysis Group (NAG) frequency quality report by the European Network of Transmission System Operators for Electricity (ENTSO-E)^54^. Table 5 shows the frequency quality parameters for the 60 Hz network as specified by ENTSO-E, including the permitted frequency ranges. The frequency recovery range allows deviations of up to $\:120\:mHz$ for 15 min, ensuring rapid stabilization and reliable operation of the power system.

**Table 5:** Frequency quality parameters of synchronous regions at 60 hz^54^.

Frequency ranges	Allowable time	State
60.12–59.88 Hz	Continuous	Standard frequency range
61.2–58.8 Hz	–	Maximum instantaneous frequency deviation
60.6–59.4 Hz	–	Maximum steady-state frequency deviation
60.6–59.4 Hz*	1 min	Frequency, and the time recovery range
60.12–59.88 Hz	15 min	Frequency, and time restore range

Notably, the assessment of the proposed methodology relied on two primary signals for all scenarios. These two signals are the WT d-axis rotor voltage $\:\left({V}_{dr}\right)$ and the SG speed deviation (∆ω). Additionally, the settling time, oscillation range (minimum and maximum overshoots), standard deviation (STD), ITSE, and ITAE were adopted in this assessment as performance indices.

The robust assessment of the coordinating WT PI-VR controller gains alongside PID-PSS and PI-type LL-PSS using both optimization algorithms, GOA and OOA, has been thoroughly conducted based on the scenarios and operating conditions. The assessment process illustrates the GOA’s capability to enhance the dynamic stability of the system by coordinating various controller configurations against the OOA. Another layer of investigation is added by assessing the GOA performance against a well-established optimization algorithm, such as PSO, which yields clear and verifiable results that are valuable for ongoing research on coordinating controllers and dynamic stability studies. The assessment also highlights the superior performance and robustness of the PI-type LL-PSS versus the PID-PSS, improving the dynamic stability of the system under study.

### Test scenario no. 1

**Fig. 11:**
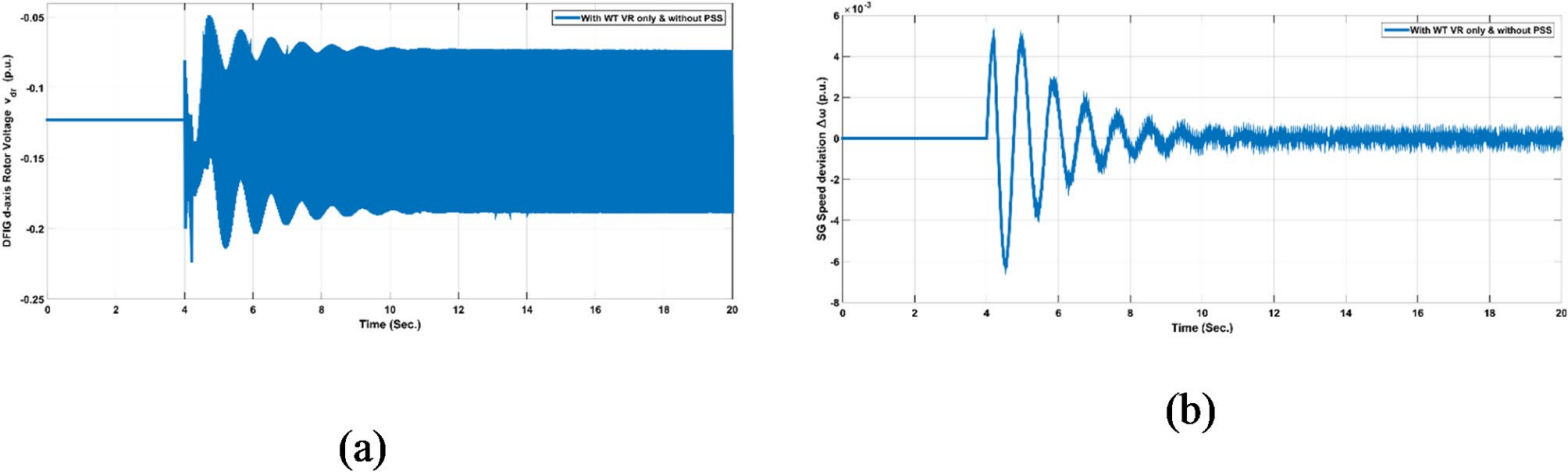
System response signals $\:{V}_{dr},\:\:and\:\varDelta\:\omega\:$ to test scenario No. 1 at operating point No. 1.

In this scenario, 30% of the system load was suddenly removed in the fourth second of the simulation period and returned after 0.1 s. The load under the test was located at the sending end of the transmission line (TL). This test was performed at two different operating points, as listed in Table 3. Notably, the test was conducted with and without coordination between the two controllers. Figure 11 shows the system response to test scenario No. 1 at operating point No. 1. Figure 11a and b depict $\:{V}_{dr},\:\:and\:\varDelta\:\omega\:$, respectively, when the WT PI-VR controller is not coordinated with the PSS controller.

The system response during test scenario No. 1, without coordination between the controllers’ gains, revealed a persistent instability state characterized by continuous oscillations. It is worth noting that test scenario No. 1 is the least severe scenario among the two scenarios considered in this study. This finding underscores that the absence of coordination between controllers leads to inherent instability, as verified by the stability analysis.

**Fig. 12:**
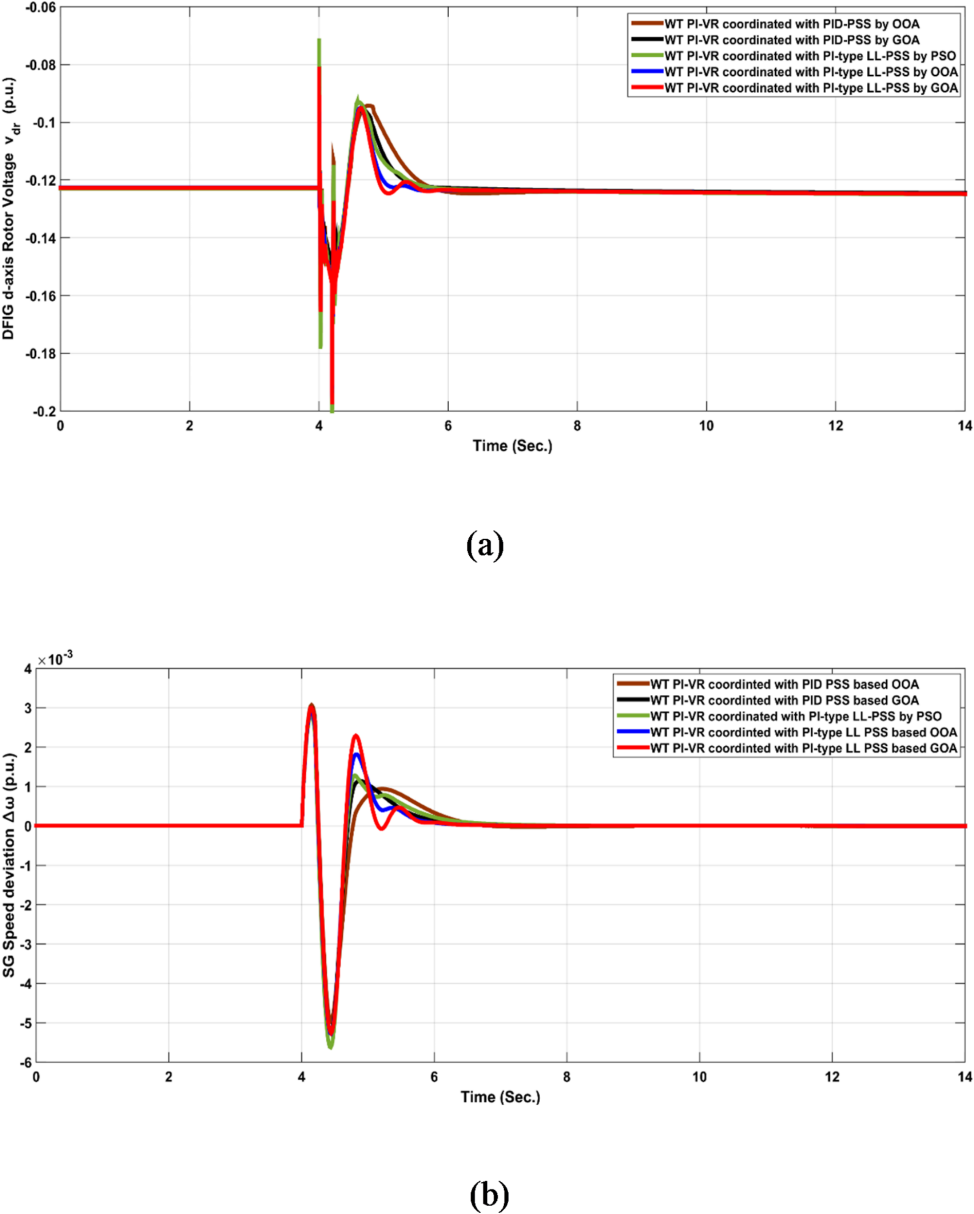
System response signals $\:{V}_{dr},\:\:and\:\varDelta\:\omega\:$ to test Scenario No. 1 at operating point No. 1 when coordinating different controllers under different configurations.

The coordinated performance of various controllers using different optimization algorithms is shown in Fig. 12. Figure 12a and b depicts $\:{V}_{dr},\:\:and\:\varDelta\:\omega\:$, respectively, when various controller gains are coordinated simultaneously using PSO, OOA, and GOA. Irrespective of the slight differences between their responses, the five-coordinated configurations successfully stabilized and damped the system oscillations rapidly compared to the controller performances without coordination. The $\:{V}_{dr}$ and $\:\varDelta\:\omega\:$ responses, as shown in Fig. 12, report the smallest maximum overshoot, ITSE, and ITAE when the gains of the WT PI-VR controller are optimized in a coordinated manner with the PI-type LL PSS using GOA. However, the WT PI-VR controller coordinated with the PI-type LL PSS based on OOA had the shortest settling time. These results ensure that the coordinated optimization of WT PI-VR with PI-type LL PSS using the GOA has superior performance compared to the other configurations.

Considering operating point No. 2 in test scenario No. 1, Table 6 summarizes the response analysis of the five configurations adopted in this study. The response analysis verified that the gains of the WT PI-VR controller were optimized in a coordinated manner with the PI-type LL PSS controller using GOA, which has robust performance compared with other configurations. This conclusion is supported by the smallest STD, ITSE, and ITAE values shown in Table 6.

**Table 6:** Analysis of the system response signals $\:{V}_{dr},\:\:and\:\varDelta\:\omega\:$ to test scenario No. 1 at operating point No. 2 under different coordination configurations.

Signal	OA	Configuration	Settling time (sec.)	(Settling max, min) range (*P*.U.)	STD	ITSE	ITAE
$\:{V}_{dr}$	OOA	WT PI-VR with PID PSS	5.8365	**(− 0.0437**, − 0.1931) 0.1494	0.01641	0.8717	9.2220
	GOA	WT PI-VR with PID PSS	5.7992	(− 0.0541, **− 0.182**) **0.1279**	0.01487	0.8786	9.2618
	PSO	WT PI-VR with PI-type LL PSS	**5.4861**	(− 0.08439, − 0.2814) 0.3658	0.03884	0.8971	9.3518
	OOA	WT PI-VR with PI-type LL PSS	**5.3185**	(− 0.0561, − 0.1842) 0.12810	0.01507	0.8833	9.2859
	GOA	WT PI-VR with PI-type LL PSS	5.4692	(− 0.0556, − 0.1845) 0.1288	**0.01481**	**0.8823**	**9.2804**
$\:\varDelta\:\omega\:$	OOA	WT PI-VR with PID PSS	6.6515	(3.632e−3, **− 5.796e−3)** 0.0094	1.224e−3	4.5567e−5	1.6845e–−2
	GOA	WT PI-VR with PID PSS	6.3335	**(3.559e−3**, − 6.065e−3) 0.0096	**1.0743e−3**	4.4542e−5	1.52289e−2
	PSO	WT PI-VR with PI-type LL PSS	6.0693	**(5.25e−3**, − 3.461e−3) **0.008891**	**1.368e−3**	3.9396e−5	1.8794e−2
	OOA	WT PI-VR with PI-type LL PSS	5.7846	(3.725e−3, − 6.221e−3) 0.0099	1.103e−3	3.8768e−5	1.4772e−2
	GOA	WT PI-VR with PI-type LL PSS	**5.7337**	(3.798e−3, − 6.236e−3) 0.0100	1.125e−3	**3.7460e−5**	**1.4642e−2**

A statistical analysis was conducted to thoroughly verify the differences in performance under various assessment scenarios. Statistical analysis relies on the average percentage of performance indices to highlight the improvement in performance for the five different controller configurations. The performance was enhanced by 51.5% when the WT PI-VR controller was coordinated optimized with PID-PSS based on GOA over OOA, and the coordinated optimization of PI-type LL-PSS and WT PI-VR was improved by 3.4% using GOA compared with OOA. The adoption of the optimized PI-type LL-PSS with WT PI-VR achieved an improvement of 25.8% compared with the PID-PSS controller. In addition, the GOA-based coordinated optimization of WT PI-VR and PI-type LL-PSS improves the system stability by 65.65% over PSO.

### Test scenario no. 2

In this scenario, a three-phase short-circuit fault was applied at the middle of the TL. The fault lasts for six cycles. Table 7 presents a response analysis of the five coordinated configurations considering operating point No. 1. This performance analysis shows the smallest STD, ITSE, and ITAE when the gains of the WT PI-VR controller are optimized in a coordinated manner with the PI-type LL PSS using GOA.

**Table 7:** An analysis of the system response signals $\:{V}_{dr},\:\:and\:\varDelta\:\omega\:$ to test Scenario No. 2 at operating point No. 1 when coordinating different controllers under different configurations.

Signal	OA	Configuration	Settling time (sec.)	(Settling max–min) range (*P*.U.)	STD	ITSE	ITAE
$\:{V}_{dr}$	OOA	WT PI-VR with PID PSS	**4.1204**	(0.09862, − 0.3209) 0.4195	0.03751	1.4770	12.0209
	GOA	WT PI-VR with PID PSS	4.1390	(0.0735, − 0.3227) **0.3962**	0.04011	1.4786	12.0235
	PSO	WT PI-VR with PI-type LL PSS	4.9226	**(0.0460**, − 0.3206) **0.3962**	0.04087	1.5059	12.0335
	OOA	WT PI-VR with PI-type LL PSS	4.2028	(0.08013, **− 0.3178)** 0.3979	0.03779	1.4814	12.0326
	GOA	WT PI-VR with PI-type LL PSS	4.4123	(0.07972, − 0.3181) 0.3978	**0.03556**	**1.46611**	**12.0128**
$\:\varDelta\:\omega\:$	OOA	WT PI-VR with PID PSS	6.5276	(3.971e−3, − 3.471e−3) 0.0076	6.944e−4	4.4531e−5	4.9720e−3
	GOA	WT PI-VR with PID PSS	5.7893	**(3.139e−3**, − 3.714e−3) 0.0078	7.372e−4	4.6549e−5	4.9648e−3
	PSO	WT PI-VR with PI-type LL PSS	6.1442	3.889e−3, − 3.85e−3) 0.0076	7.352e−4	1.2982e−5	6.9115e−3
	OOA	WT PI-VR with PI-type LL PSS	5.5945	(4.034e−3, **− 3.086e−3)** **0.0071**	9.728e−4	1.2755e−5	6.9030e−3
	GOA	WT PI-VR with PI-type LL PSS	**5.5110**	(4.036e−3, − 3.291e−3) 0.0073	**6.799e−4**	**1.2529e−5**	**4.2833e−3**

**Fig. 13:**
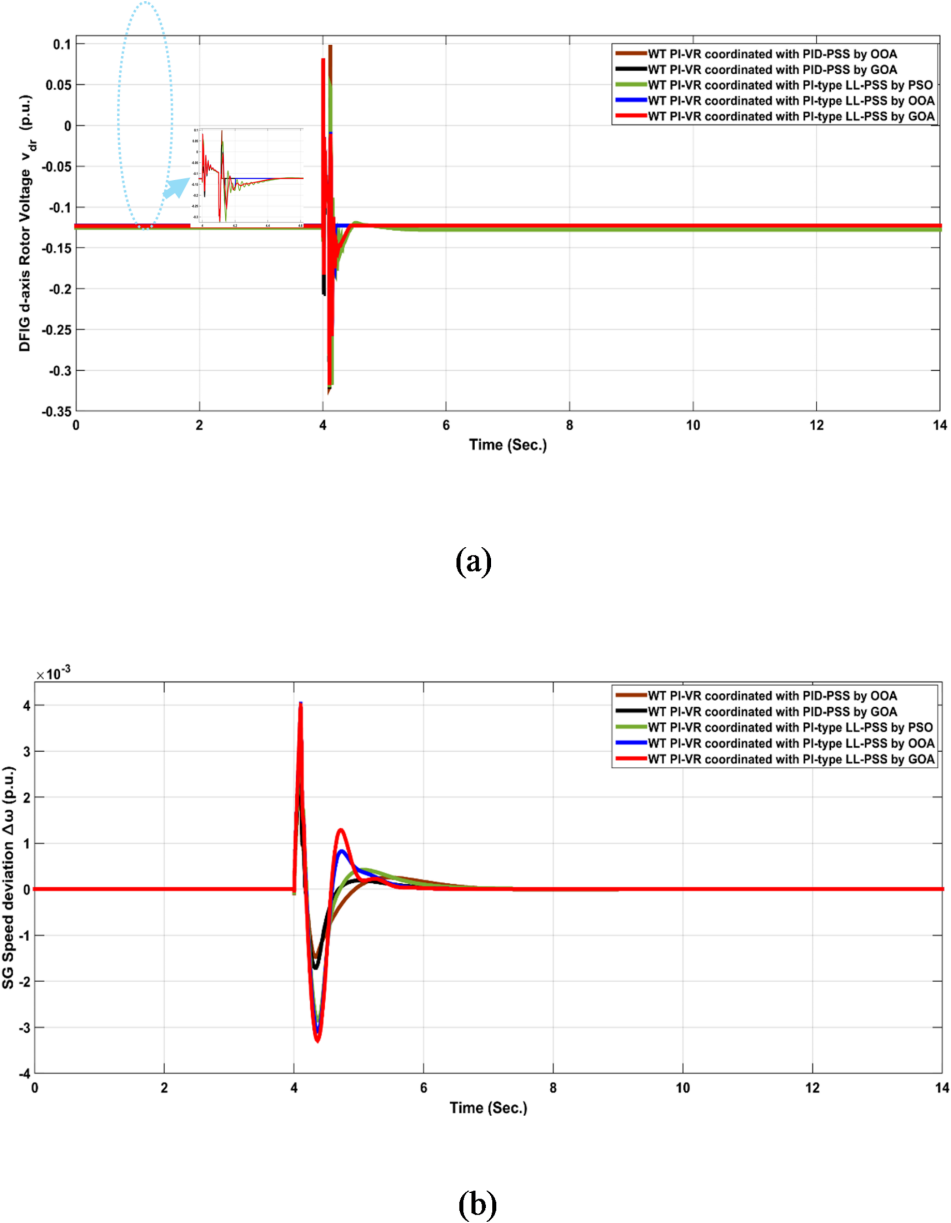
System response signals $\:{V}_{dr},\:\:and\:\varDelta\:\omega\:$ to test scenario No. 2 at operating point No. 2 when coordinating different controllers under different configurations.

Figure 13 shows the system’s response with five coordinated configurations at operating point No. 2. GOA-based coordinated optimization achieved robust performance compared with other optimization algorithms with PI-type LL PSS.

**Fig. 14:**
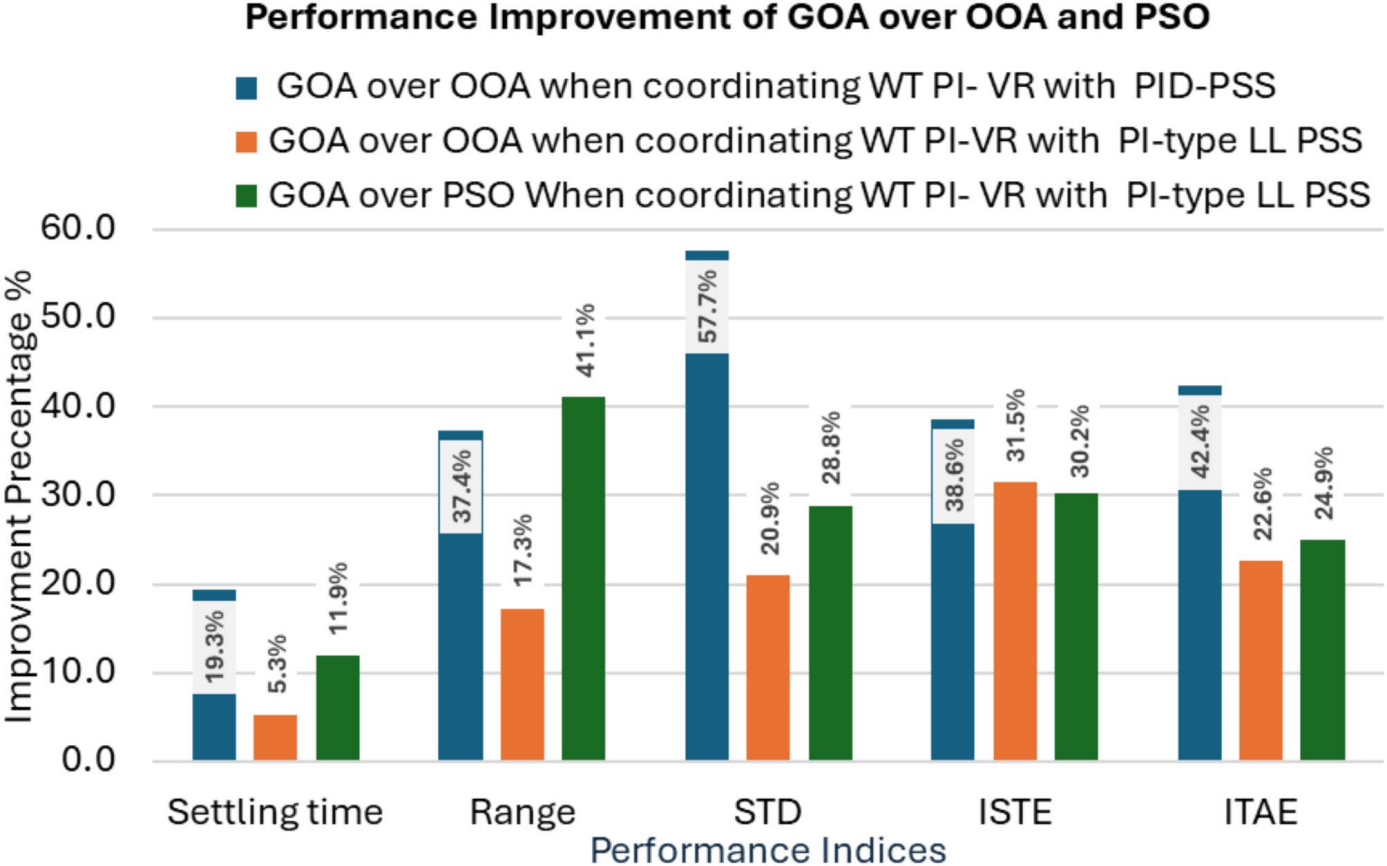
Average percentage improvement between different coordinating configurations.

**Fig. 15:**
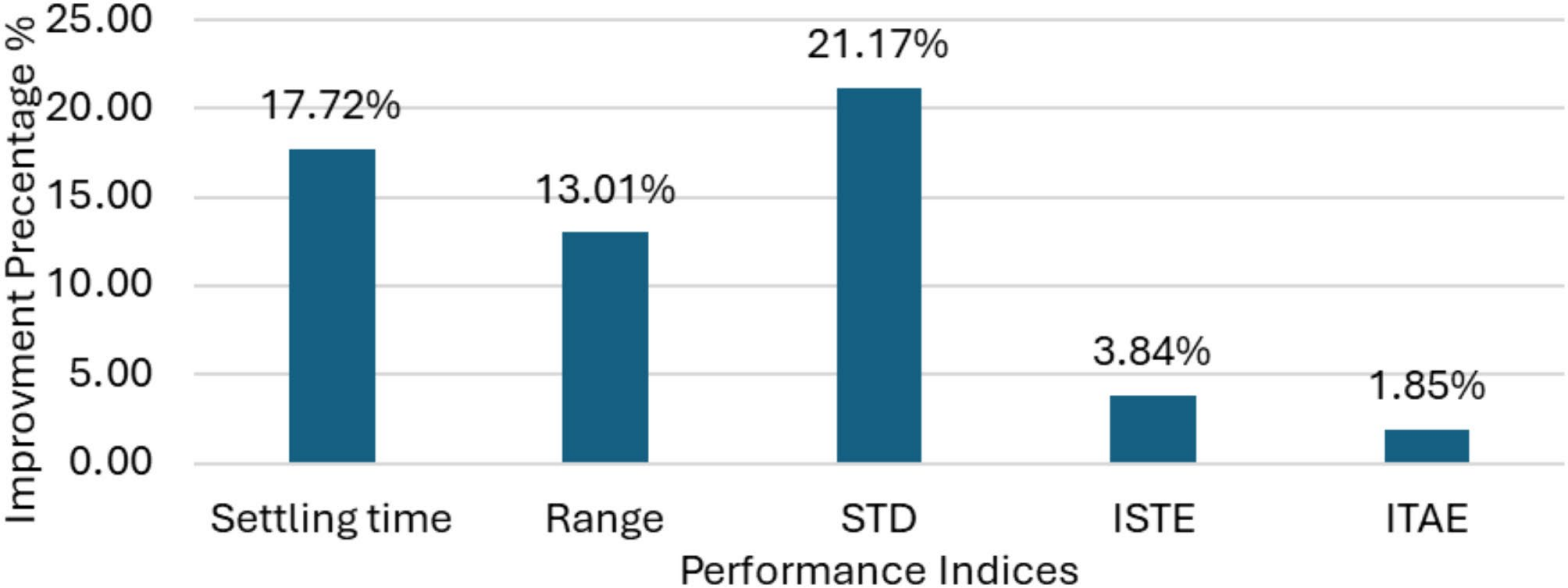
Average percentage outperformance of PI-type LL-PSS compared to PID-PSS under different format configurations.

Like test scenario No. 1, statistical analysis was conducted. The performance of the WT PI-VR in conjunction with PID-PSS controllers based on the GOA improved 34.3% compared with the OOA. GOA-based coordinated optimization of WT PI-VR and PI-type LL-PSS reported an enhancement of 22.4% in performance over OOA. However, the PID-based controller in this case reports an improvement of 14.6% compared to the PI-type LL-PSS, which experiences a larger settling time. Notably, GOA-based coordinated optimization improved the system stability of approximately 71.35% compared to PSO. Figure 14 summarizes the average percentage improvement for different configurations. Figure 15 shows the performance of both controller structures adopted for the PSS in this study.

In a nutshell, the system stability improved by 48.85% when adopting the GOA to optimize the gains of the WT PI-VR with PID-PSS controllers. Additionally, the GOA-based coordinated optimization of the PI-type LL-PSS and WT PI-VR enhanced the system stability by 24.40% compared with OOA. Compared with a well-established PSO, the GOA reported a significant improvement of approximately 34.23% compared with the PSO. This enhanced performance owing to the adoption of the GOA emphasizes its capability to offer a robust coordinated optimization of the PI controller. Finally, the performance of the PI-type LL-PSS with WT PI-VR was more robust than that of the PID-PSS. This improvement was estimated at approximately 14.4%, as shown in Fig. 15.

Table 8 shows a quantitative comparative study of the proposed methodology compared to other state-of-the-art and recent optimization and coordination techniques.

**Table 8:** A quantitative comparison of the proposed methodology with recent state-of-the-art coordination techniques.

Study	Optimization algorithm	Control strategy	Performance improvement (%)	Settling time reduction (%)	ITAE reduction (%)	Tested scenarios
Lenine et al. (2024)^55^	Predictive voltage control	Interlinking converter for microgrids	36.5%	22.1%	18.3%	Power sharing stability
Prakasa et al. (2024)^56^	Modified Harris Hawk optimization	PSS & virtual inertia control	41.2%	28.7%	19.6%	Frequency response
Nigam (2024)^57^	Adaptive SPS	Smart protection system (SPS)	30.8%	20.3%	17.2%	Relay coordination
Djalal et al. (2024)^58^	Mayfly optimization algorithm	Multi-band PSS3C for wind farms	45.3%	31.6%	20.5%	Small-signal stability
Djalal (2025)^56^	AI-based optimization	FACTS-based MB-PSS control	38.9%	26.4%	21.1%	NA
Satapathy et al. (2024)^59^	–	Hydro-PSS coordination	34.7%	23.5%	18.9%	NA
Proposed technique	GOA	WT PI-VR & PI-type LL-PSS coordination	48.85% (GOA vs. OOA)	34.23% (GOA vs. PSO)	24.40% (GOA vs. OOA)	Step change, 3-phase fault

Table 8 illustrates the effectiveness of the GOA in enhancing power system stability by coordinating the WT VR with PSS. This proposed method achieves an impressive stability improvement of 48.85%, significantly surpassing existing approaches summarized in the table such as Mayfly Optimization (45.3%)^58^, Modified Harris Hawk Optimization (41.2%)^56^, and FACTS-based Model-Based Power System Stabilizer control (38.9%)^58^. Additionally, the GOA reduces the settling time by 34.23%, making it more efficient than conventional optimization techniques like PSO and OOA. Regarding ITAE reduction, the GOA displays a 24.40% improvement over OOA when using a PI-type LL-PSS, indicating enhanced damping and transient stability.

Furthermore, while most previous studies have primarily focused on improvements in single test scenario, the proposed approach has been validated under various scenarios, including step changes and three-phase short circuits. This demonstrates its robustness against different grid disturbances. These results confirm that the GOA outperforms existing optimization techniques and offers a more comprehensive solution for enhancing dynamic stability in renewable-integrated power systems.

## Conclusions

This study introduces a coordinated optimization approach for Power System Stabilizers (PSS) of synchronous generators and Wind Turbine Voltage Regulators (WT VR) using the goose Optimization Algorithm (GOA) to enhance power system stability. Different controller structures (PI vs. PID) robustness is assessed under various disturbances. Simulation results emphasize the excellence of simultaneously optimizing controller gains. Additionally, the results analysis shows that coordinating WT PI-VR with PI-type LL-PSS significantly improves stability compared to coordinating WT PI-VR with PID-PSS. Key performance indices, including settling time, ITAE, ISTE, standard deviation, and oscillation range, are used in this study. The detailed analysis of performance indices confirms the robustness and effectiveness of the proposed controller coordination strategy.

Quantitatively, detailed analysis showed that the GOA-based approach achieved a 48.85% stability improvement over OOA when optimizing PID-PSS, and a 24.40% improvement when applied to PI-type LL-PSS. Coordinating WT PI-VR with PI-type LL-PSS improved stability by 14.4% compared to PID-PSS, demonstrating superior damping capabilities. The proposed method also outperformed PSO, achieving a 34.23% improvement in stability metrics.

These results highlight the potential of GOA-based coordination for enhancing long-term stability, reducing sensitivity to external disturbances, and improving dynamic response. The proposed method is scalable and applicable to power systems incorporating diverse renewable energy sources. However, further investigation is warranted in several areas, including: (1) the integration of additional renewable energy resources such as photovoltaic (PV) systems and fuel cells, (2) the application of the proposed approach to multimachine systems and microgrids, and (3) the impact of communication-induced time delays on system performance.

## Electronic supplementary material

Below is the link to the electronic supplementary material.

Supplementary Material 1

## Data Availability

The datasets used and/or analyzed during the current study are available from the corresponding author upon reasonable request.

## **Appendixes**

### Appendix (A)

For the MSMIB power system data.

- DIFG WT data (10 MVA).
  $$\:{R}_{S}=0.00706\:p.u.$$, $$\:{Ll}_{S}=0.171\:p.u.$$, $$\:{R}_{r}^{{\prime\:}}=0.005\:p.u.$$, $$\:{Ll}_{r}^{{\prime\:}}=0.156\:p.u.$$, $$\:{L}_{m}=2.9\:p.u.$$, $$\:{H}_{g}=5.04\:Sec$$,$$\:R=33.05\:m$$,
  $$\:\rho\:=1.204\:\raisebox{1ex}{$kg$}\!\left/\:\!\raisebox{-1ex}{${m}^{3}$}\right.,\:{C}_{p}=0.4800,\:{X}_{tg}=0.3\:p.u.,\:{X}_{S}=0.02\:p.u.$$
- SG data (10 MVA).
  $$\:{X}_{d}=1.6507\:p.u.$$, $$\:{X}_{d}^{{\prime\:}}=0.2543\:p.u.$$, $$\:{X}_{d}^{{\prime\:}{\prime\:}}=0.2641\:p.u.$$, $$\:{X}_{q}=1.5921\:p.u.$$, $$\:{X}_{q}^{{\prime\:}}=0.2119\:p.u.$$, $$\:{X}_{q}^{{\prime\:}{\prime\:}}=0.1409\:p.u.$$,
  $$\:{T}_{d0}^{{\prime\:}}=4.536\:Sec.$$, $$\:{T}_{d0}^{{\prime\:}{\prime\:}}=0.0424\:Sec.,\:{T}_{q0}^{{\prime\:}}=0.6776\:Sec.$$, $$\:{T}_{q0}^{{\prime\:}{\prime\:}}=0.0433\:Sec.$$, $$\:H=3.7\:Sec.$$, $$\:{X}_{l}=0.1409\:p.u.$$
- SG Exciter.
  $$\:{K}_{a} = 200,\:{T}_{a} = 0.001\:Sec.,\:{T}_{r} = 0.02 \, s.,\:{E}_{fdmin}=0,\:{E}_{fdmax}=7$$
- Transmission System.
  $$\:{l}_{TL}=100\:km,\:{R}_{TL}=0.01755\:{\Omega\:}/km\:,\:{L}_{TL}=0.8737\:mH/km$$

## List of symbols

${\Delta S}_{m}$: Synchronous generator (SG) speed deviation, in P.U.
${T}_{ms}, {T}_{m}$: SG and Wind Turbine (WT) mechanical torque, respectively, in P.U.
$\delta$: SG rotor angle, in rad.
${S}_{m}$: SG rotor speed, in P.U.
${E}{\prime}$: SG voltage behind q-axis transient reactance, in P.U.
${V}_{ref}$: SG reference voltage, in P.U.
$i$: SG generated current, in P.U.
${X}_{t1}$: Inductive reactance of the transformer connects SG and the power system, in P.U.
${V}_{t}, {V}_{BC}$: Voltage at bus A, and bus C, respectively, in P.U.
${v}_{W}$: Wind speed, in P.U.
${\omega }_{r}$: WT rotor electrical angular speed, in P.U.
${v}_{r},{i}_{r}$: Double-fed induction generator (DFIG) rotor voltage and current, respectively, in P.U.
${V}_{s}, {i}_{s}$: DFIG stator voltage and rotor current, respectively, in P.U.
${V}_{s}, {i}_{s}$: DFIG stator voltage and rotor current, respectively, in P.U.
${X}_{tg}$: Inductive reactance of the transformer connects the feed to the DFIG rotor circuit in P.U
${v}_{g}, {i}_{g}$: Grid side converter voltage and current, respectively, in P.U.
${V}_{DC}$: DFIG DC-link voltage, in P.U.
${i}_{dr}^{*}$: DFIG voltage regulator output current, in P.U.
${R}_{e1}, {X}_{l}$: The resistance and inductive reactance of the T.L. connecting WT to the grid, respectively, in P.U.
${i}_{e}$: WT generated current, in P.U.
${X}_{e}$: The T.L. inductive reactance connecting Bus A and the infinite bus, in P.U.
${L}_{m}$: DFIG magnetizing inductance, in P.U.
${i}_{ds}, {i}_{qs}$: DFIG d-q axes stator currents, respectively, in P.U.
${i}_{dr}, {i}_{qr}$: DFIG d-q axes rotor currents, respectively, in P.U.
$\rho$: Air Density, in kg.m2.
$\beta$: WT pitch angle, in deg.
$R$: WT rotor radius, in m.
${C}_{p}$: DFIG power coefficient of the WT rotor blades
${\omega }_{t}$: Angular speed of WT blades, in P.U.
${v}_{d}, {v}_{q}$: SG d-q axes terminal voltages, respectively, in P.U.
${i}_{d}, {i}_{q}$: SG d-q axes stator currents, respectively, in P.U.
$\Delta {e}_{d}{\prime}, \Delta {e}_{q}{\prime}$: SG transient emf induced deviation in d-q axes, respectively, in P.U.
${X}_{d}, {X}_{q}$: SG Stator d-q axes inductive reactance, respectively, in P.U.
${e}_{fd}$: SG field or exciter output voltage, in P.U.
${K}_{a}$: SG AVR gain.
$\Delta {x}_{1}$: Effects of dynamic deviation on field voltage, in P.U.
${T}_{es}, {T}_{e}$: SG and WT electrical torque, respectively, in P.U.
${X}_{d}{\prime}, {X}_{q}{\prime}$: SG d-q axes transient reactance, respectively, in P.U.
${e}_{bd},{e}_{bq}$: d-q axes voltage at the infinite bus, respectively, in P.U.
${i}_{de}, {i}_{qe}$: d-q axes currents through WT integration, respectively, in P.U.
${\omega }_{b}$: Base angular frequency, in P.U.
${X}_{tg}$: Transformer inductive reactance connecting the grid side converter, in P.U.
${v}_{dg}, {v}_{qg}$: Grid side converter d-q axes voltages, respectively, in P.U.
${v}_{D}$: Voltage magnitude dependent on the slope reactance, in P.U.
$${X}_{S}$$: Slope reactance (P.U./$${P}_{norm}$$)
${K}_{PV}, {K}_{IV}$: WT voltage regulator optimized gains
${v}_{d}^{{^{\prime}}}$: Voltage output signal of the current regulator
${v}_{d}^{*}$: Modified control signal to the rotor side converter
${R}_{r}$: DFIG rotor winding resistance
${L}_{lr}$: DFIG rotor winding leakage inductance
${\upomega }_{0}$: Synchronous angular speed.
${\upomega }_{\text{r}}$: DIFG rotor electrical angular speed
${\mathbf{X}}_{Gi}(t+1)$: Position of the goose at iteration (t).
${\mathbf{X}}_{Gbest}(t)$: best position of the goose.
${\mathbf{X}}_{Gj}\left(t\right), {{\varvec{X}}}_{Gk}\left(t\right)$: Positions of randomly selected geese
α, β, and γ: Balance and foraging factors
${\mathbf{X}}_{Gi}(0)$: Initial position of $ith$ goose.
${\mathbf{X}}_{\text{Gmax}}, {\mathbf{X}}_{\text{Gmin}}$: Lower and upper bounds of the search space, respectively.
$e\left(t\right)$: Error signal to be measured

## References

[1] Banupriya, R., Nagarajan, R. & Muthu Balaji, S. Power electronic converters in the unbalance compensation method for renewable energy-powered active distribution systems: AOA-RERNN approach. *Soft Comput.* **28**, 10583–10600. 10.1007/S00500-024-09853-2/TABLES/8 (2024).

[2] Wu, Y. et al. Inertia enhancement for doubly fed induction generators with comprehensive inertia control and enhanced phase-locked loop regulation. *Electr. Power Syst. Res.* **238**, 111201. 10.1016/j.epsr.2024.111201 (2025).

[3] Bahgat, M., Ezzat, M., Attia, M. A., Mekhamer, S. F. & Elbehairy, N. M. Comparative analysis of PI and fuzzy logic controller for grid connected wind turbine under normal and fault conditions. *Sci. Rep.* **15**, 1954. 10.1038/s41598-024-85073-w (2025). 39809924 10.1038/s41598-024-85073-w PMC11733149

[4] Arora, A., Bhadu, M. & Kumar, A. Simultaneous power Oscillation damping and frequency control in AC microgrid considering renewable uncertainties: A coordinated control of multiple robust controllers with imperfect communication. *Iran. J. Sci. Technol. Trans. Electr. Eng.* **48**, 165–185. 10.1007/s40998-023-00649-y (2024).

[5] Araújo-de-Oliveira, J. D., de-Araújo-Lima, F. K., Nogueira, F. G., Tofoli, F. L. & Branco, C. G. C. Stability analysis and application of a synchronverter-based control approach to DFIG-based wind energy conversion systems. *Electr. Power Syst. Res.* **232**, 110403. 10.1016/j.epsr.2024.110403 (2024).

[6] Khalid, M. Smart grids and renewable energy systems: perspectives and grid integration challenges. *Energy Strategy Reviews*. **51**, 101299. 10.1016/J.ESR.2024.101299 (2024).

[7] Nader, M. A., Ibrahim, Hossam, E. A., Talaat, A. M. & Shaheen, Bassam, A. Hemade. Optimization of power system stabilizers using Proportional-Integral-Derivative Controller-Based antlion algorithm: experimental validation via electronics environment. *Sustainability* **15**, 8966. 10.3390/su15118966 (2023).

[8] Hindocha, B. R. & Sheth, C. V. Improving the stability and damping of low-frequency oscillations in grid-connected microgrids with synchronous generators. *Electr. Eng.* **106**, 4881–4901. 10.1007/s00202-024-02257-3 (2024).

[9] Hossain, M. S. et al. Improvement of low-frequency Oscillation damping in power systems using a deep learning technique. *Eng. App AI*. **137**, 109176. 10.1016/j.engappai.2024.109176 (2024).

[10] Abedini, M. A novel controller algorithm to improve stability of power system based on a hybrid of fuzzy controller and Gray Wolf optimization by coordinating PSS and TCSC with considering uncertainty. *Soft Comput. 2024*. **28**, 23. 10.1007/S00500-024-10369-Y (2024).

[11] Ibrahim, N. M.A., Elnaghi, B. E., Ibrahim, H. A. & Talaat, H. E. A. Performance assessment of bacterial foraging based power system stabilizer in multi-machine power system. *Int. J. Intell. Syst. Appl.* **11**, 43–53. 10.5815/ijisa.2019.07.05 (2019).

[12] Sarkar, D. U. & Prakash, T. A convolutional neural network framework to design power system stabilizer for damping oscillations in multi-machine power system. *Neural Comput. Appl.* **36**, 5059–5075. 10.1007/s00521-023-09323-0 (2024).

[13] Tadj, M. et al. Improved chaotic Bat algorithm for optimal coordinated tuning of power system stabilizers for multimachine power system. *Sci. Rep.* **14**, 15124. 10.1038/s41598-024-65101-5 (2024). 38956387 10.1038/s41598-024-65101-5 PMC11219771

[14] Zhang, C., Chang, X., Dai, J., Chen, Z. & Babanezhad, M. Designing of a wide-area power system stabilizer using an exponential distribution optimizer and fuzzy controller considering time delays. *Sci. Rep.* **15**, 1773. 10.1038/s41598-025-85524-y (2025). 39800722 10.1038/s41598-025-85524-y PMC11725599

[15] Yang, Y. et al. Parameter coordination optimization of power system stabilizer based on similarity index of power system state-BP neural network. *Energy Rep.* **9**, 427–437. 10.1016/j.egyr.2023.04.158 (2023).

[16] Ibrahim, N.M.A., El-said, E. A.Attia, H. E. M. & Hemade, B. A. Enhancing power system stability: an innovative approach using coordination of FOPID controller for PSS and SVC FACTS device with MFO algorithm. *Electr. Eng.*10.1007/s00202-023-02051-7 (2023).

[17] He, P. et al. Coordinated optimization of PSS and STATCOM-POD based on LHS-MCSM to improve probabilistic small-signal stability of power system. *Electr. Eng.*10.1007/s00202-024-02763-4 (2024).

[18] Ramya, R., Suganyadevi, M. V. & Usha, S. Design and performance analysis of multiobjective optimization using PSO and SVM for PSS tuning in SMIB system. *Industrial Control Syst.*10.1002/9781119829430.ch6 (2024).

[19] Nader, M. A., Ibrahim, B. E., Elnaghi, Hamed, A., Ibrahim, Hossam, E. A. & Talaat Modified particle swarm optimization based on Lead-Lag power system stabilizer for improve stability in Multi-Machine power system. *Int. J. Electr. Eng. Inf.* **11**, 161–182. 10.15676/ijeei.2019.11.1.10 (2018).

[20] Kar, M. K. Stability analysis of multi-machine system using FACTS devices. *Int. J. Syst. Assur. Eng. Manage.* **14**, 2136–2145. 10.1007/s13198-023-02044-6 (2023).

[21] Vijaya Lakshmi, A. S. V., Siva Kumar, M. & Ramalinga Raju, M. Interval approach based decentralized robust PID-PSS design for an extended Multi-machine power system. *Arab. J. Sci. Eng.* **49**, 6293–6304. 10.1007/s13369-023-08197-7 (2024).

[22] Barik, S. K. & Mohapatra, S. K. Coordinated control of multi band PSS (MBPSS) with type 2 fuzzy based SSSC controller design for power system stability improvement. *Evo Intel*. **17**, 1909–1932. 10.1007/s12065-023-00871-x (2024).

[23] Tae, D. & Tamma, K. K. A robust and generalized effective DAE framework encompassing different methods, algorithms, and model order reduction for linear and nonlinear second order dynamical systems. *Finite Elem. Anal. Des.* **228**, 104043. 10.1016/j.finel.2023.104043 (2024).

[24] Shah, N. N. & Joshi, S. R. Small signal stability analysis of DFIG based wind farms connected to SMIB. Conf. on Smart Grids, Power and Advan. Con. Eng., ICSPACE 2017, vol. 2018- January, 2017. (2017). Inter 10.1109/ICSPACE.2017.8343437

[25] Bhukya, J. Enhancing the wind farm-based power system stability with coordinated tuned supplementary controller. *Int. J. Circuit Theory Appl.* **51**. 10.1002/cta.3705 (2023).

[26] Surinkaew, T. & Ngamroo, I. Coordinated robust control of DFIG wind turbine and Pss for stabilization of power oscillations considering system uncertainties. *IEEE Trans. Sustain. Energy*. **5**. 10.1109/TSTE.2014.2308358 (2014).

[27] Bhukya, J., Naidu, T. A., Vuddanti, S. & Konstantinou, C. Coordinated control and parameters optimization for PSS, POD and SVC to enhance the transient stability by integrating DFIG based wind power systems. *Int. J. Emerg. Electr. Power Syst.* **23**. 10.1515/ijeeps-2021-0098 (2022).

[28] Shah, N. N. & Joshi, S. R. Utilization of DFIG-based wind model for robust damping of the low frequency oscillations in a single SG connected to an infinite bus. *Int. Trans. Electr. Energy Syst.* **29**. 10.1002/etep.2761 (2019).

[29] Dasu, B., Mangipudi, S. & Rayapudi, S. Small signal stability enhancement of a large scale power system using a bio-inspired Whale optimization algorithm. *Prot. Control Mod. Power Syst.* **6**. 10.1186/s41601-021-00215-w (2021).

[30] Satapathy, S., Nahak, N., Patra, A. K., Mishra, A. K. & Technology Steam governor based optimal power oscillation damping controller with solar penetrations. In *2023 International Conference in Advances in Power, Signal, and Information APSIT 2023*, 379–384. 10.1109/APSIT58554.2023.10201667

[31] Ranka, T. et al. GMTO primary mirror active support system testing with the test cell and mass simulator. **13094**, 598–618. (2024). 10.1117/12.3020281

[32] Satapathy, S., Nahak, N., Mishra, A. K., Patra, A. K. & Memetic, S. S. A. Tuned compensated hydro governor to damp power system oscillations for variable wind penetrations. *Lecture Notes Mech. Eng.*, 583–594. 10.1007/978-981-97-1080-5_48/FIGURES/10 (2024).

[33] Nahak, N., Sahoo, S. R. & Mallick, R. K. Small signal stability enhancement of power system by modified GWO-optimized UPFC-based PI-lead-lag controller. *Adv. Intell. Syst. Comput.* **817**. 10.1007/978-981-13-1595-4_21 (2019).

[34] Barik, S. K., Mohapatra, S. K. & Patra, A. K. Coordinated control of STATCOM based controller using de Algorithm. In *International Conference on Computational Intelligence for Smart Power System and Sustainable Energy, CISPSSE 2020*. 10.1109/CISPSSE49931.2020.9212225 (2020).

[35] Nahak, N., Praharaj, K. & Mishra, A. Dynamic stability enhancement of a variable solar penetrated power system by fractional upfc-based controller. *Lect. Not. Electr. Eng.* **661**. 10.1007/978-981-15-4692-1_10 (2021).

[36] Hamad, R. K. & Rashid, T. A. GOOSE algorithm: a powerful optimization tool for real-world engineering challenges and beyond. *Evol. Syst.* **15**. 10.1007/s12530-023-09553-6 (2024).

[37] Trojovský, P., Trojovská, E. & Akbari, E. Economical-environmental-technical optimal power flow solutions using a novel self-adaptive wild geese algorithm with stochastic wind and solar power. *Sci. Rep.* **14**. 10.1038/s41598-024-54510-1 10.1038/s41598-024-54510-1 PMC10876935 38374395

[38] Bento, M. E. C. Load margin assessment of power systems using recurrent neural network and greylag goose optimization. *IFAC-Papers OnLine* **58**, 656–661. 10.1016/j.ifacol.2024.07.587 (2024).

[39] Song, D., Yin, M., Chen, Z., Zhou, L. & Zou, Y. Maximum power point tracking control of wind turbines based on speed hysteresis loop to reduce drive-train loads. *Electr. Power Syst. Res.* **238**, 111110. 10.1016/j.epsr.2024.111110 (2025).

[40] Pani, S. R. & Samal, R. K. Evaluation of reliability and resilience in wind integrated power systems using 80 meter mast measurements. *Electr. Power Syst. Res.* **241**, 111263. 10.1016/J.EPSR.2024.111263 (2025).

[41] Xu, Y., Chi, Y. & Yuan, H. Stability-constrained optimization for modern power system operation and planning. In *Stability-Constrained Optimization for Modern Power System Operation and Planning*, 1–464. 10.1002/9781119848899

[42] Emami, A., Araújo, R., Cruz, S., Hadla, H. & Aguiar, A. P. A systematic approach to modeling synchronous generator using Markov parameters and Takagi–Sugeno fuzzy systems. *Expert Syst. Appl.* **235**, 121122. 10.1016/J.ESWA.2023.121122 (2024).

[43] Doleski, O. D. & Müller, M. F. Handbook of electrical power systems: energy technology and management in dialogue. *Springer Nat. Switz.*10.1515/9783111264271 (2024).

[44] Djamila, R. Wind power electric systems: modeling, simulation, control, and power management control. *Springer Nat. Switz.*10.1007/978-1-4471-6425-8 (2024).

[45] Shah, N. N. & Joshi, S. R. Small signal stability analysis of DFIG based wind farms connected to SMIB. *2017 Int. Conf. Smart Grids Power Adv. Control Eng. ICSPACE 2017*. **2018**, 247–252. 10.1109/ICSPACE.2017.8343437 (2017).

[46] Starke, G. M., Meneveau, C., King, J. R. & Gayme, D. F. A dynamic model of wind turbine Yaw for active farm control. *Wind Energy*. **27**, 1302–1318. 10.1002/WE.2884 (2024).

[47] Vardhan, A. S. S. & Sinha, U. K. Control strategies and performance analysis of doubly fed induction generator for grid-connected wind energy conversion system. *Electr. Eng.* **106**, 1203–1224. 10.1007/S00202-023-02079-9/FIGURES/31 (2024).

[48] Paiva, P. & Castro, R. Effects of battery energy storage systems on the frequency stability of weak grids with a high-share of grid-connected converters. *Electronics* **13**, 1083. 10.3390/ELECTRONICS13061083 (2024).

[49] Aoun, S., Boukadoum, A. & Yousfi, L. Advanced power control of a variable speed wind turbine based on a doubly fed induction generator using field-oriented control with fuzzy and neural controllers. *Int. J. Dyn. Control*. **12**, 2398–2411. 10.1007/S40435-023-01345-9/TABLES/11 (2024).

[50] Hamad, R. K. & Rashid, T. A. GOOSE algorithm: a powerful optimization tool for real-world engineering challenges and beyond. *Evol. Syst.* **15**, 1249–1274. 10.1007/S12530-023-09553-6/TABLES/24 (2024).

[51] Boopathi, D., Jagtheesan, K., Samanta, S. & Naidu, K. Performance analysis of a Multi-objective Function-Based PID controller for. *Syst. Freq. Regul.* **2024**, 115–132. 10.1007/978-981-97-0353-1_6

[52] Jabari, M., Ekinci, S., Izci, D., Bajaj, M. & Zaitsev, I. Efficient DC motor speed control using a novel multi-stage FOPD(1 + PI) controller optimized by the pelican optimization algorithm. *Sci. Rep. 2024*. **14**, 1. 10.1038/s41598-024-73409-5 (2024). 10.1038/s41598-024-73409-5 PMC11438883 39341933

[53] Nayak, P. C., Prusty, R. C. & Panda, S. Adaptive fuzzy approach for load frequency control using hybrid moth flame pattern search optimization with real time validation. *Evol. Intell.* **17**, 1111–1126. 10.1007/S12065-022-00793-0/FIGURES/15 (2024).

[54] Erik, Ø. et al. *NAG - Frequency Quality Report, Phase 2* (The European Network of Transmission System Operators for Electricity (ENTSO-E), 2017).

[55] Sai Sampath Kumar, P., Suresh, P. & Lenine, D. Performance improvement of predictive voltage control for interlinking converters of integrated microgrid. *Measurement: Sens.* **33**, 101196. 10.1016/J.MEASEN.2024.101196 (2024).

[56] Prakasa, M. A., Robandi, I., Borghetti, A., Djalal, M. R. & Himawari, W. Coordinated design of power system stabilizer and virtual inertia control using modified Harris Hawk optimization for improving power system stability. *IEEE Access.*10.1109/ACCESS.2024.3522291 (2024).

[57] Renewable Energy Integration in Utility Grids. Advances in Power Quality. - Google Books n.d. accessed February 22, (2025). https://books.google.com.eg/books?hl=en&id=-8IAEQAAQBAJ&redir_esc=y

[58] Ruswandi Djalal, M., Robandi, I. & Almas Prakasa, M. Stability improvement of Sulselrabar system with integrated wind power plant using Multi-Band PSS3C based mayfly optimization algorithm. *IEEE Access.* **12**, 76707–76734. 10.1109/ACCESS.2024.3406434 (2024).

[59] Satapathy, S., Nahak, N. & Patra, S. A new integrated modelling and control of ternary pumped storage hydro power generation for small signal stability studies. *J. Energy Storage*. **103**, 114179. 10.1016/J.EST.2024.114179 (2024).

