# Supplementary material for: Enhancing power system stability by coordinating a wind turbine voltage regulator and lead-lag power system stabilizer using GOOSE optimization
Source: Sci Rep. 2025 Apr 30;15:15242. doi: 10.1038/s41598-025-97419-z (PMC12043978; doi:10.1038/s41598-025-97419-z)
Supplement: Supplementary file 1 — Supplementary Material 1 [file 41598_2025_97419_MOESM1_ESM.docx]

**Appendix (A)**

For the MSMIB power system data.

- DIFG WT data (10 MVA)

$R_{S}=0.00706 p.u.$, ${Ll}_{S}=0.171 p.u.$, $R_{r}^{'}=0.005 p.u.$, ${Ll}_{r}^{'}=0.156 p.u.$, $L_{m}=2.9 p.u.$, $H_{g}=5.04 Sec$,$R=33.05 m$,

$\rho=1.204 \frac{kg}{m^{3}}$, $C_{p}=0.4800$, $X_{tg}=0.3 p.u.$, $X_{S}=0.02 p.u.$

- SG data (10 MVA)

$X_{d}=1.6507 p.u.$, $X_{d}^{'}=0.2543 p.u.$, $X_{d}^{''}=0.2641 p.u.$, $X_{q}=1.5921 p.u.$, $X_{q}^{'}=0.2119 p.u.$, $X_{q}^{''}=0.1409 p.u.$,

$T_{d0}^{'}=4.536 Sec.$, $T_{d0}^{''}=0.0424 Sec., T_{q0}^{'}=0.6776 Sec.$, $T_{q0}^{''}=0.0433 Sec.$, $H=3.7 Sec.$, $X_{l}=0.1409 p.u.$

- SG Exciter

$K_{a}=200$, $T_{a}=0.001 Sec.$, $T_{r}=0.02Sec.$, $E_{fdmin}=0$, $E_{fdmax}=7$

- Transmission System

$l_{TL}=100 km$, $R_{TL}=0.01755 \Omega/km$, $L_{TL}=0.8737 mH/km$
